# Domain-Dependent Evolution Explains Functional Homology of Protostome and Deuterostome Complement C3-Like Proteins

**DOI:** 10.3389/fimmu.2022.840861

**Published:** 2022-03-10

**Authors:** Maoxiao Peng, Zhi Li, João C. R. Cardoso, Donghong Niu, Xiaojun Liu, Zhiguo Dong, Jiale Li, Deborah M. Power

**Affiliations:** ^1^ Key Laboratory of Exploration and Utilization of Aquatic Genetic Resources, Ministry of Education, Shanghai Ocean University, Shanghai, China; ^2^ Comparative Endocrinology and Integrative Biology, Centre of Marine Sciences, Universidade do Algarve, Faro, Portugal; ^3^ Shanghai Engineering Research Center of Aquaculture, Shanghai Ocean University (SHOU), Shanghai, China; ^4^ Co-Innovation Center of Jiangsu Marine Bio-industry Technology, Jiangsu Ocean University, Lianyungang, China; ^5^ Department of Biotechnology and Biomedicine, Yangtze Delta Region Institute of Tsinghua University, Jiaxing, China; ^6^ Shanghai Ocean University International Center for Marine Studies, Shanghai, China

**Keywords:** conserved domain, functional homologues, parallel evolution, complement C3/C4/C5 family, C3-like protein a-subunit

## Abstract

Complement proteins emerged early in evolution but outside the vertebrate clade they are poorly characterized. An evolutionary model of C3 family members revealed that in contrast to vertebrates the evolutionary trajectory of *C3-like* genes in cnidarian, protostomes and invertebrate deuterostomes was highly divergent due to independent lineage and species-specific duplications. The deduced *C3-like* and vertebrate C3, C4 and C5 proteins had low sequence conservation, but extraordinarily high structural conservation and 2-chain and 3-chain protein isoforms repeatedly emerged. Functional characterization of three *C3-like* isoforms in a bivalve representative revealed that in common with vertebrates complement proteins they were cleaved into two subunits, b and a, and the latter regulated inflammation-related genes, chemotaxis and phagocytosis. Changes within the thioester bond cleavage sites and the a-subunit protein (ANATO domain) explained the functional differentiation of bivalve *C3-like*. The emergence of domain-related functions early during evolution explains the overlapping functions of bivalve *C3-like* and vertebrate C3, C4 and C5, despite low sequence conservation and indicates that evolutionary pressure acted to conserve protein domain organization rather than the primary sequence.

## Introduction

The complement system is of central importance for immunity and complement 3 (*C3*) is at the core of its function (1–3). C3 belongs to the thioester bond containing protein (TEP) superfamily (4) that also includes α_2_-macroglobulin (A2M) (5), pregnancy zone protein (PZP) (6), CD109 (7) and PZP-like A2M domain-containing 8 (CPAMD8) (8). The appearance and differentiation of C3, A2M and CD109 occurred after the divergence of the sponges and before the divergence of cnidaria from the bilaterian lineage (9, 10). The *C3* gene has been identified in all deuterostomes studied so far (1). Studies directed at deciphering the evolution of the complement system suggest a common ancestral molecule gave rise to *C3* and to two others complement molecules *C4* and *C5* in vertebrates (11–15). The *C3* prototype gene underwent several rounds of duplication before the cyclostome (lampreys and hagfish) divergence and in the vertebrates *C3*, *C4* and *C5* gene members emerged (11, 13). Evolution and function of the complement pathway in the species rich protostome clade is less well resolved (16–20).

The complement system in vertebrates straddles the innate and acquired immune response. It is activated by three pathways [the classical, alternative and mannose binding lectin (MBL)] and all converge on *C3* (21, 22). Complement activation causes proteolytic cleavage of C3, C4 and C5 and generates a smaller anaphylatoxin “a” protein subunit and larger “b” subunit in vertebrates. The end point of the cascade is the formation of the membrane attack complex (MAC, C5bC6C7C8C9) that causes cell lysis (2, 23–25).

We hypothesized that the non-vertebrate prototype *C3* gene, designated *C3-like* in this study, is the functional homologue of vertebrate *C3*, *C4* and *C5* genes. Available evidence indicates that complement activation *via* both the lectin and alternative pathways is possible in protostomes and emerged in a similar evolutionary timeframe in cnidarians (10, 26–29). MAC complex homologues (C6 and C7/C8/C9) have not been identified in protostomes and the main activity assigned to complement is opsonization (30). Nonetheless, genes encoding MAC-type domain containing proteins (MACPF) have been identified in a marine gastropod, the periwinkle (*Littorina littorea*) (31). Moreover, gastropod and bivalve hemolymph has cytotoxic activity (32) and duplicate *C3-like* (*C3-like-1* and *C3-like 2*) genes with hemolytic activity have been identified in the Chinese razor clam (*Sinonovacula constricta*) (14, 33, 34).

Exploiting the burgeoning availability of genomes and transcriptomes for cnidarian, protostomes and invertebrate deuterostomes we establish a model for complement evolution focused on *C3-like* genes and reveal a common origin with vertebrate genes but with highly divergent evolutionary trajectories. The primary amino acid sequence of the deduced C3-like and vertebrate C3, C4 and C5 proteins has low sequence conservation but a well conserved domain structure. Homologues of vertebrate two-chain structural isoforms typical of mature C3 and C5 proteins (composed of α and β subunits) and 3-chain isoforms typical of mature C4 protein (with α, β and γ subunits) (3, 35) repeatedly emerged in the protostomes and other non-vertebrate phyla and share common activities.

## Results

### *C3-Like* Genes Evolved by Lineage and Species-Specific Events

**Figure 1:**
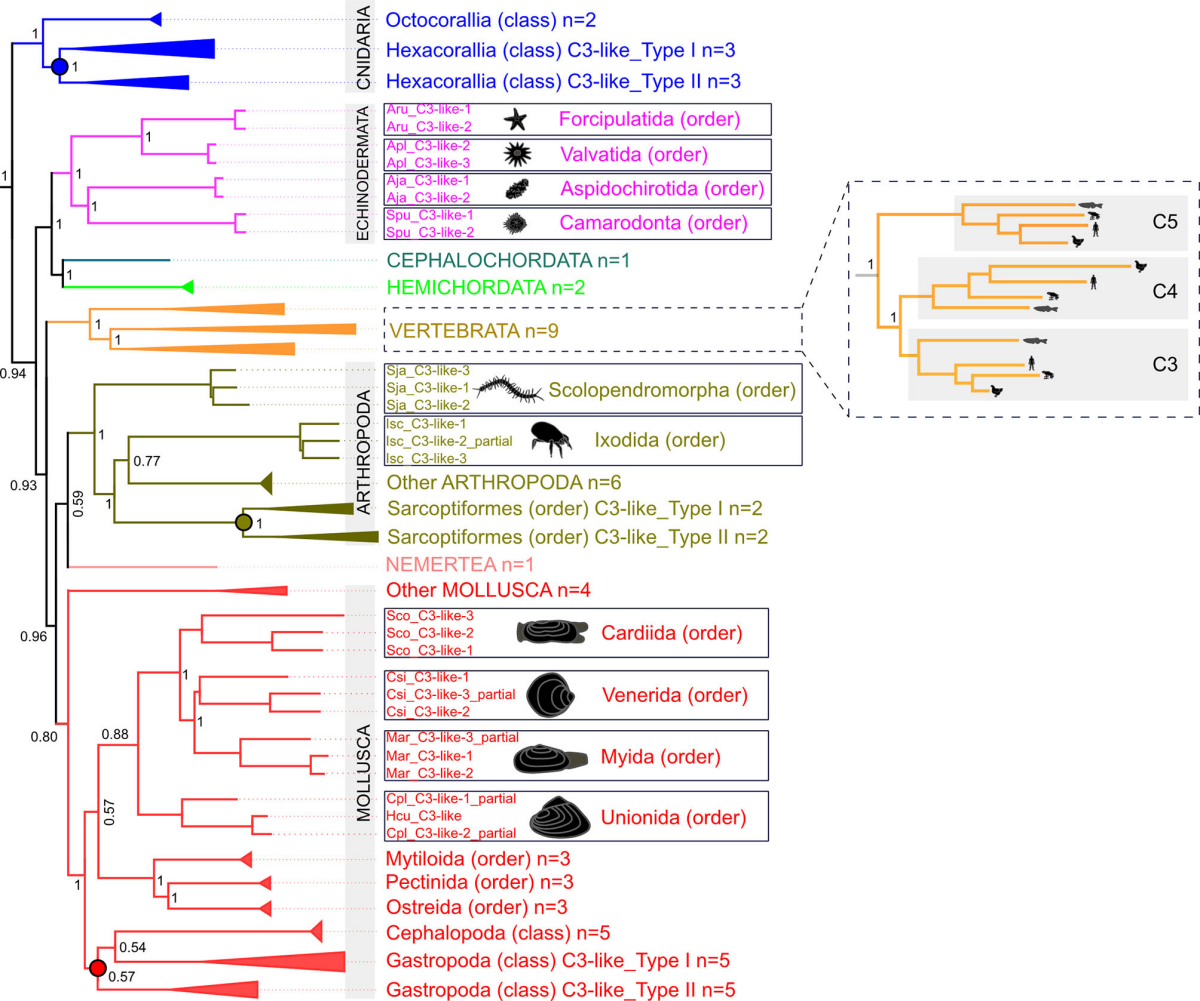
Collapsed phylogenetic tree of the cnidaria, protostomes and invertebrate deuterostomes *C3-like* genes. The phylogenetic tree was constructed using the Bayesian Inference (BI) method and posterior probability values for the main branches are represented. Sequences from 56 species of diverse non-vertebrate phyla and with representatives of different orders and classes were used. Analysis included C3, C4 and C5 from 9 vertebrates for comparisons. To facilitate visualization some branches were collapsed. The tree was rooted with human CD109 and A2MG but these sequences are not shown in the figure. The unedited phylogenetic tree is available as **Supplementary Figure 1A**. Branches with different colors indicate the species of different phyla: Cnidarian (deep blue), Nemertean (pink), Mollusca (red), Arthropoda (olive green), Echinodermata (purple), Cephalochordata (green), Hemichordata (light blue) and Vertebrata (orange). Circles in the branches indicate lineage-specific duplication events. The boxes highlight the sequences that arose by species-specific events and a silhouette of the different animals is presented. The number of species (n) collapsed within each tree branch is indicated. An ML tree with a similar topology is available as **Supplementary Figure 1B**. The source and accession numbers of the sequences used are in **Supplementary Table 1**.

Database searches identified *C3-like* genes in 56 species representatives of different phyla (5 Cnidaria, 1 Nemertea, 31 Mollusca, 12 Arthropoda, 4 Echinodermata, 2 Hemichordata, 1 Cephalochordata) and revealed that gene number was variable across species. Phylogenetic analysis (**Figure 1** and **Supplementary Figure 1** **)** suggested that the metazoan *C3-like* ancestral molecule underwent distinct evolutionary trajectories in different phyla. Although since most sequences were obtained from transcriptomes and not genomes it was unclear if all forms of *C3-like* that exist were retrieved for all species (**Supplementary Table 1**). No clustering with the vertebrate *C3*, *C4* and *C5* was found but phyla specific clustering of the modern *C3-like* genes occurred and the diverse gene number in cnidaria, protostomes and invertebrate deuterostomes resulted from: a) lineage-specific and b) species-specific duplications events. Clustering of the invertebrate deuterostomes (Hemichordata, Echinodermata and Cephalochordata phyla) *C3-like* genes suggest that evolution was similar to the protostome model, but their early radiation prior to the protostome and vertebrate clades explains the greater gene sequence divergence (**Figure 1** and **Supplementary Figure 1** **)**. The *C3-like* gene first emerged in the Cnidaria phylum but was subsequently lost from representative species of the phyla of Annelida, Dicyemida, Platyhelminthes, Nematoda and in some subphylum of Arthropoda (Hexapoda and Crustacea). Lineage duplication events led to multiple *C3-like* genes in Cnidaria (Hexacorallia, subclass), Arthropods (Ixodes and Sarcoptiformes orders) and Mollusca (Gastropoda class) phyla generating two types of *C3-like* (Type I and Type II). Species-specific duplications of the *C3-like* gene were also identified in species of the Arthropoda (Scolopendromorpha and Ixodida order), Mollusca (Cardiida, Venerida, Myida and Unionida orders) and in the deuterostome Echinodermata (Forcipulatida, Valvatida, Aspidochirotida, Camarodonta orders) phyla (**Figure 1**).

### C3-Like Proteins Share Conserved Sequence Motifs and Similar Protein Chains With Vertebrates

**Figure 2:**
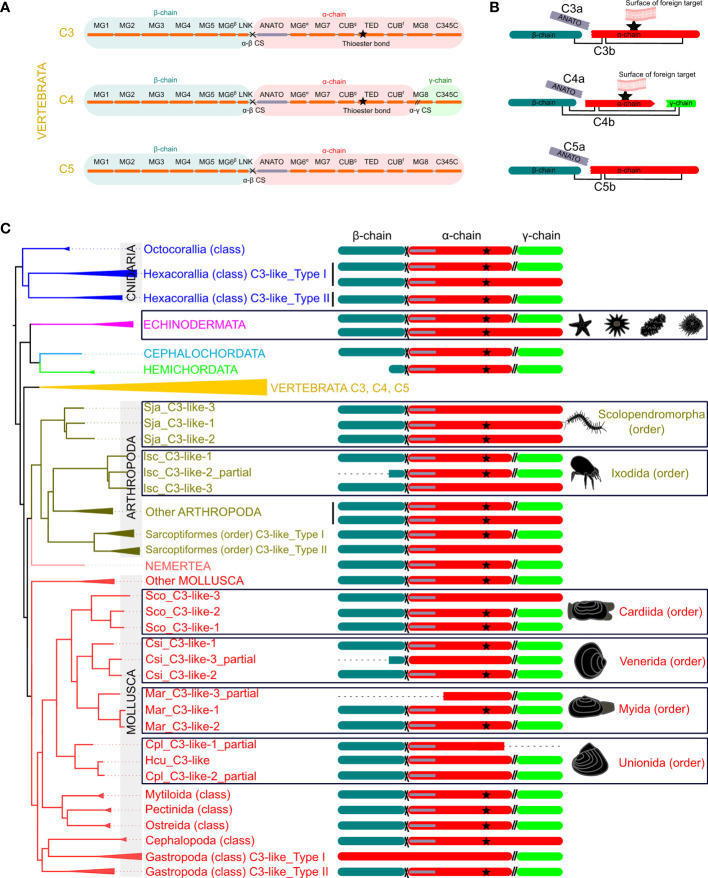
Schematic representation of the protein domains of the vertebrate C3, C4 and C5 and the non-vertebrate C3-like deduced proteins. **(A)** Linear representation of the human C3, C4 and C5 structure with the localization of the 13 domains elucidated from the crystallographic structure of human C3 (3, 36, 37) [eight MG- macroglobulin domains (MG1-8); the link domain (LNK), anaphylatoxin domain (ANATO), the complement C1r/C1s, Uegf, Bmp1 domain (CUB), thioester-containing domain (TED) and the carboxy-terminal domain (C345C)]; the α-β (cross symbol) and α-γ (parallel lines symbol), cleavage sites and the three protein chains (β (blue), α (red) and γ (green) -chain). **(B)** Predicted mature protein structure of human C3, C4 and C5. The two mature protein subunits, “a” (only with the ANATO domain) and “b” for each protein is represented. The star represents the thioester bond, and the connecting lines represent the disulphide bridges that connect the different protein chains. **(C)** Mature protein structure predicted for C3-like. Only the predicted chains and ANATO domains, cleavage sites and thioester bond are represented. The dashed line represents incomplete sequences. The phylogenetic tree represented is a simplified version of **Figure 1** and branches with different colors indicate different phyla. The deduced structure of the C3-like proteins that are proposed to have emerged from species-specific duplication events are boxed to highlight their predicted structural diversity. The detailed figure with all species and protein motifs is available in **Supplementary Figure 2A**. The source and accession numbers of the sequences are given in **Supplementary Table 1**.

**Figure 3:**
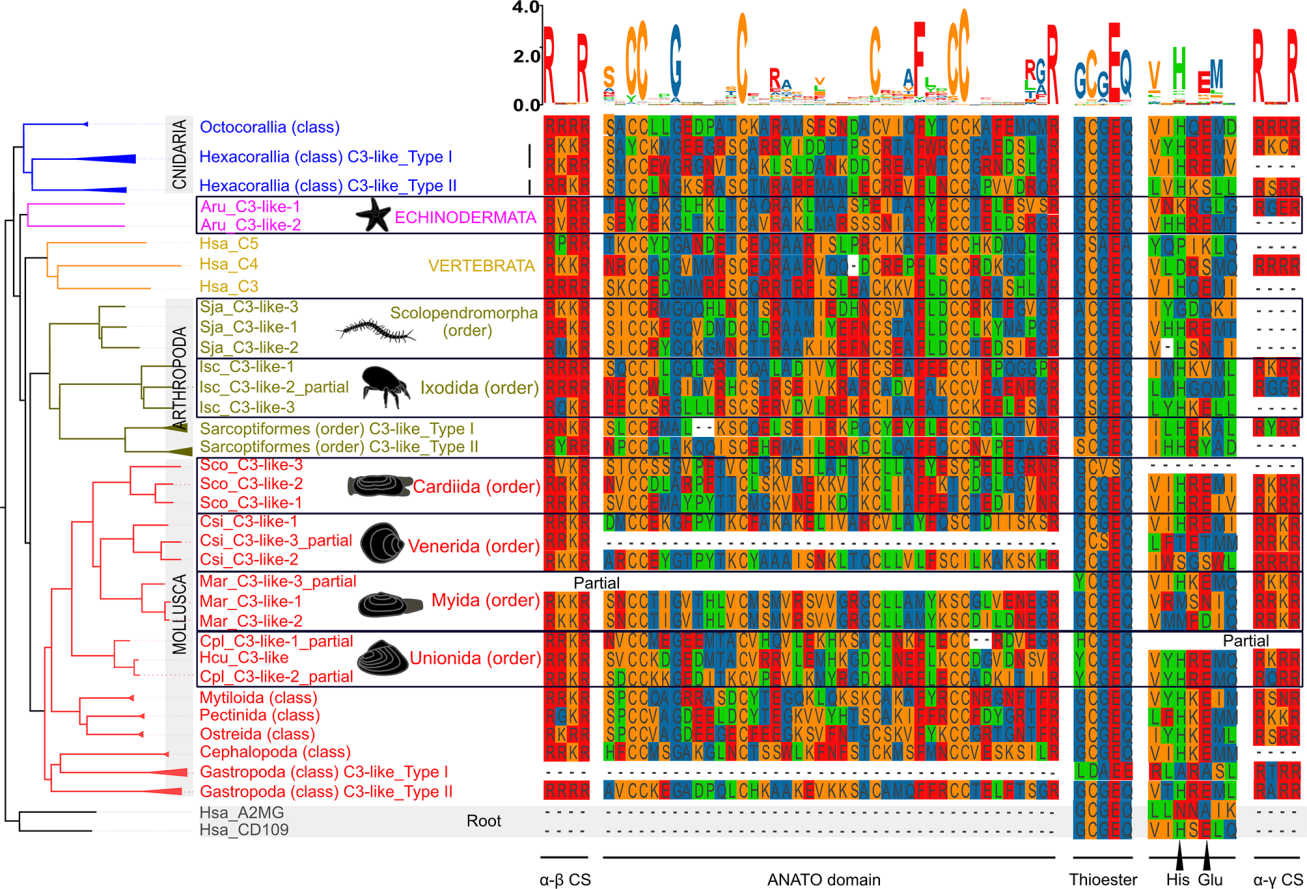
Sequence conservation of the C3-like functional domains and motifs. The alignment of the predicted amino acid sequences for the ANATO domain (a-subunit), the thioester bond and His-Glu amino acid residues essential for the binding of C3 and C4 to the surface of foreign targets, and the two cleavage sites: α-γ(CS) and α-β are represented. This figure is a summary version of the full figure available in **Supplementary Figure 2B**. The different protein domains and motifs represented were deduced from the C3-like sequence alignment containing all collected sequences. Amino acids were colored using the MSA default settings and letters of different heights are indicative of amino acid residue conservation across sequences (the bigger the letter, the higher the sequence conservation). The dashed line indicates the regions where no sequence overlap was found. The sequences of the Echinodermata, Arthropoda and Mollusca species-specific C3-like proteins are boxed. The ANATO domain represented is the automatic trimmed sequence alignment of the most conserved regions using the Multiple alignment trimming tool with a site coverage cut-off= 0.95 in the TBtools software (38). The source and accession numbers of the sequences used are listed in **Supplementary Table 1**.

The 13 characteristic domains of the vertebrate C3, C4 and C5 proteins (3, 36, 37) such as the eight macroglobulin domains (MG1-8); the link domain (LNK), anaphylatoxin domain (ANATO), the complement C1r/C1s, Uegf, Bmp1 domain (CUB), the thioester-containing domain (TED) and the carboxy-terminal domain (C345C) are present in the deduced cnidaria, protostome and invertebrate deuterostome C3-like proteins. The deduced protein structure of C3-like also contained the two enzymatic cleavage sites (α-β and α-γ) (**Figure 2** and **Supplementary Figure 2A**) responsible for the release of β, α and γ protein chains that generate the functional structure. More specifically the “RXXR” motif (where X is K, P, Q, M or T) in the α-β cleavage site and the α-γ cleavage site was highly conserved in C3-like from Cnidaria and other phyla up to the vertebrate C3, C4 and C5 (**Figure 2**). The α-γ cleavage site was absent from some forms of C3-like for example in the Cnidarian, the cauliflower coral *Pocillopora damicornis* C3-like-2 (PdaC3-like-2), in the Arthropoda, the Chinese red-headed centipede *Scolopendra japonica* C3-like-1 (SjaC3-like-1), in the Mollusca, the Chinese razor clam *S. constricta* C3-like-3 (ScoC3-like-3) and in the Echinodermata, the sea urchin *Strongylocentrostus purpuratus* C3-like-1 (SpuC3-like-1). In the ANATO domain, six cysteine (Cys) residues and a C-terminal arginine (Arg) residue which are responsible for the activity of the vertebrate protein a-subunit were highly conserved from Cnidaria C3-like up to vertebrate C3, C4 and C5 (**Figure 3** and **Supplementary Figure 2B**). But species-specific *C3-like* gene duplication events in Mollusca and Arthropoda produced a gene isoform with a non-conserved thioester “GCGEQ” bond analogous to what occurs in vertebrate C5. In the gastropods, *C3-like* type I gene encoded a protein that lost the α-β cleavage site, the ANATO domain and had a non-conserved thioester bond.

### Mollusca Duplicate C3-Like Isoform Function

The function of protostome *C3-like* isoforms was explored in, a bivalve mollusk (the Chinese razor clam) and their highly conserved domain structure across protostomes favors functional inference. Three *C3-like* gene members exist in the Chinese razor clam (*ScoC3-like-1* to *3*) and arose by species specific gene duplications and their deduced protein structure contained the 13 characteristic motifs, and the conserved α-β (^665^RVKR^668^) cleavage site and the ANATO domain with six conserved cysteine (Cys) residues (14, 33) (**Figure 3** and **Supplementary Figure 2B**). In ScoC3-like-3, the thioester bond structure (^1038^GCVSQ^1042^) was poorly conserved (**Figure 3** **)**. ScoC3-like-1 and ScoC3-like-2 deduced proteins had the three-chain structure (α-β cleavage site and α-γ cleavage site) typical of vertebrate C4 and ScoC3-like-3 had the two-chain structure (α-β cleavage site only) typical of vertebrate C5. All three *C3-like* genes in the Chinese razor clam had a similar tissue distribution and were present in liver, hemocytes, siphon, mantle, foot, gill, and gonad (**Supplementary Figure 3A**). *ScoC3-like-3* was significantly more abundant (p < 0.001) in the liver compared to *ScoC3-like-1* and *ScoC3-like-2*.

### Cleavage of Bivalve C3-Like Into “a and b” Functional Subunits

ScoC3-like circulates in the hemolymph (**Supplementary Figure 4** **).** To confirm if ScoC3-like is cleaved into two subunits (a-subunit and b-subunit) as occurs for vertebrate C3, native ScoC3-like-1, ScoC3-like-2 and ScoC3-like-3 in LPS activated razor clam hemolymph (SCH) were analyzed by Mass Spectrometry (MS). The C3-like peptide sequences obtained confirmed the presence of the N-terminal a-subunit cleavage site (^661^RRKR^664^, ^661^RRKR^664^ and ^665^RVKR^668^ in ScoC3-like-1, ScoC3-like-2 and ScoC3-like-3, respectively) and the C-terminal site (^754^VNR^756^, ^754^VNR^756^ and ^760^RNR^762^ was identified in ScoC3-like-1, ScoC3-like-2 and ScoC3-like-3, respectively) (**Supplementary Table 2** **)**. The theoretical molecular weight of the a-subunit for each ScoC3-like protein isoform (**Supplementary Table 3** **)** matched the predicted size of the deduced protein sequence indicating that in razor clam the three C3-like isoforms are cleaved, to generate functional “a” and “b” subunits that circulate in SCH.

### Functional Divergence of Bivalve C3-Like Subunits

The function of the two C3-like protein subunits (a and b) of the three different razor clam C3-like isoforms was characterized.

### C3-Like-b Subunit

**Figure 4:**
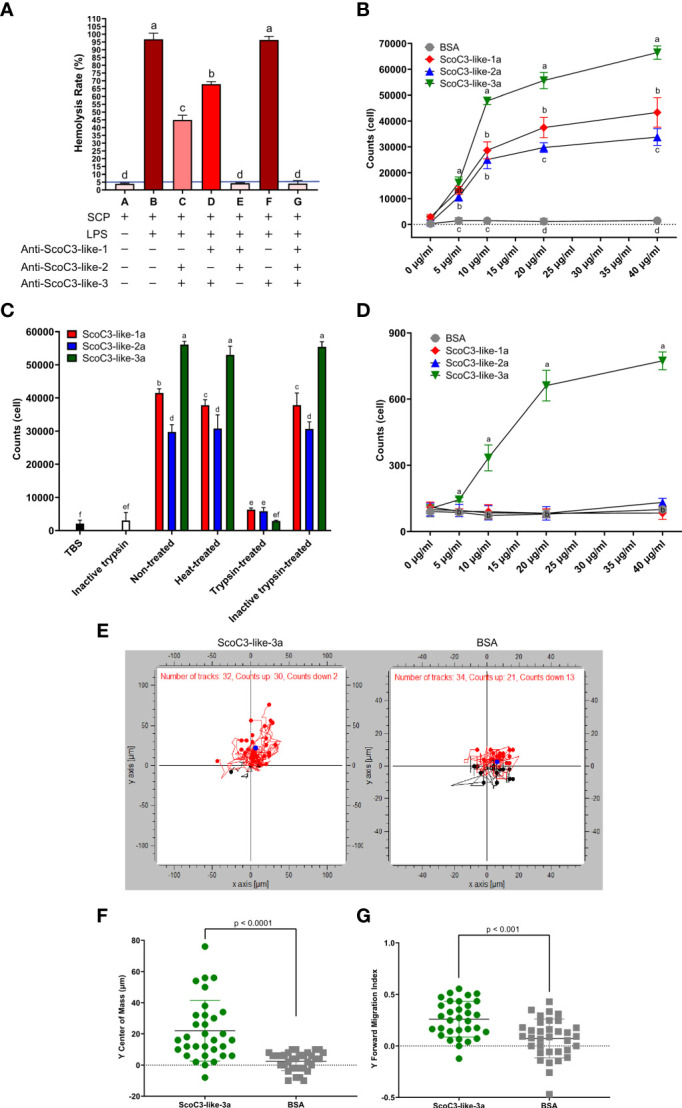
Razor clam C3-like protein functional assays. **(A)** Hemolytic activity. Rabbit erythrocytes were used. The blue line represents the threshold of hemolysis. Results are shown as the mean ± the standard error (SEM, n = 3 assays). “+” means the reagent was added and “-” means the reagent was not added. The increase in color tone of the bars represents the intensity of hemolysis. One-Way Anova was used, and different letters indicate the significantly different groups (p < 0.001). **(B)** Chemotaxis response of SCHC to recombinant *S. constricta* a-protein subunits. Values represent the mean ± SEM (n = 3 assays). Different letters indicate significantly different groups (p < 0.05). **(C)** Effect of heat and trypsinization of recombinant *S. constricta* a-protein subunits. Bars represent the mean ± SEM (n = 3 assays). One-Way Anova was used, and different letters indicate significantly different groups (p < 0.05). **(D)** Effect of recombinant ScoC3-like-a subunits on chemotaxis of J774A.1 cells. BSA was used as the positive control. Values represent the mean ± SEM (n = 3 assays). One-Way Anova was used and groups with different letters are significantly different (p < 0.05). **(E)** Migration of J774A.1 cells in response to ScoC3-like-3a. Dark points represent the center of mass (COM), red dots represent the upwards-migrating cells (red lines) and blue dots the downwards-migrating cells (black lines). BSA was used as the negative control. The chemotaxic factors (ScoC3-like-3a or BSA) were added to the lower chamber. The number of cells for which migration was tracked using the automatic setting is indicated: C3-like-3a (exposed group, n=32) and BSA (control group, n=34). **(F)** Scatter plot of the displacement of center of mass (COM) of the J774A.1 cells. Values represent the mean ± SEM (n = 32, automatic detection setting). A students t-test was used to identify significant differences between groups. The p value for the pairwise comparison is shown. **(G)** Scatter plot of the forward migration index (FMI) of the J774A.1 cells. Values represent the mean ± SEM (n = 34, automatic detection setting). A student t-test was used to identify significant differences between groups. The p value for the pairwise comparison is shown.

A hemolysis-inhibition assay with antisera specific for each ScoC3-like-b isoform was used to determine, which b-subunits provoked significant hemolysis of rabbit erythrocytes (**Figure 4A**). Antisera specific for ScoC3-like-3b did not significantly modify the hemolysis rate (p > 0.05). Antisera specific for ScoC3-like-1b (33) and ScoC3-like-2b subunits (14) both caused a significant (p < 0.05) reduction in the hemolysis rate. Addition of anti-ScoC3-like-1b serum and anti-ScoC3-like-2b serum to the hemolytic positive control (B) ablated hemolysis, revealing that ScoC3-like-1b and ScoC3-like-2b were hemolytic factors but ScoC3-like-3b was not.

### C3-Like-a Subunit

Cell chemotaxis assays and phagocytosis assays were used to characterize the function of the C3-like protein a-subunits. Chemotaxis assays using either razor clam hemocytes (SCHC) or mammalian J774A.1 cells revealed that the recombinant ScoC3-like-3a was the most active protein isoform in both assays. In the SCHC assay all recombinant ScoC3-like-a forms significantly stimulated cell migration (p < 0.001) but ScoC3-like-3a was significantly more active (p < 0.001) than the other ScoC3-like a- subunits (**Figure 4B**). Heat-treatment of ScoC3-like-a protein subunits did not significantly modify their capacity to promote SCHC migration and ScoC3-like-3a was still significantly more active (p < 0.001) (**Figure 4C** **)**. Treatment of all recombinant ScoC3-like-a protein subunits with trypsin ablated their action.

ScoC3-like-3a was the only protein tested that significantly induced J774A.1 cell chemotaxis (p < 0.001) **(** **Figure 4D**). The induced trajectory of J774A.1 cell chemotaxis towards ScoC3-like-3a and BSA (**Figure 4E**) was 94% and ~62%, respectively. The greater number of J774A.1 cells that migrated towards ScoC3-like-3a, was revealed by the significant upward shift (p < 0.0001) of the center mass [COM (blue circle)](**Figure 4F** **)** and by the induced (p < 0.001) Forward Migration Index (FMI) on the Y axis (**Figure 4G**).

**Figure 5:**
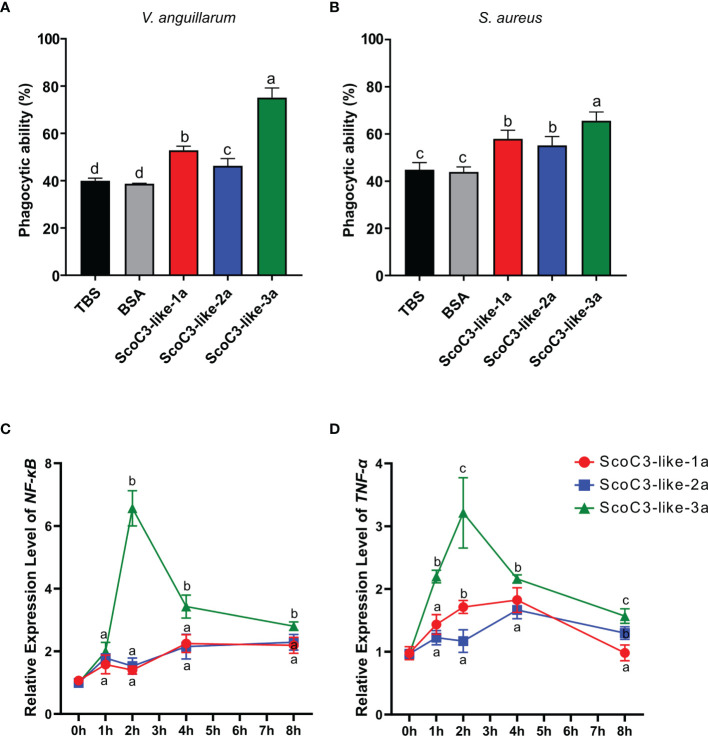
Capacity of the recombinant ScoC3-like*-*a subunit to induce SCHC phagocytic activity and their role in the regulation of the expression of immune inflammatory factors. For the phagocytosis assays two types of bacteria were used **(A)** *V. anguillarum* and **(B)** *S. aureus* and analysis were performed by flow cytometry. One-Way Anova was used and the groups with different letters are significantly different (p < 0.05). Quantitative expression analysis of NF-κB **(C)** and TNF-α **(D)** in Chinese razor clam hemocytes after exposure to recombinant ScoC3-like-a protein subunits. Relative expression level is expressed as fold-change in comparison to the control groups (TBS and BSA treated group). Values represent the mean ± SEM (n = 3 pools composed of 3 individuals/pool). One-Way Anova was performed, and different letters represent significant differences (p < 0.05) in relation to the control.

The SCHC phagocytic activity against *Staphylococcus aureus* and *Vibrio anguillarum* revealed that all recombinant ScoC3-like a-protein subunits significantly increased (p < 0.05) phagocytosis compared to the negative control (TBS) and positive control (bovine serum albumin BSA) (**Figures 5A, B** and **Supplementary Figure 5**). SCHC phagocytic ability was significantly higher in the presence of ScoC3-like-3a (p < 0.001 and p < 0.05) compared to ScoC3-like-1a and ScoC3-like-2a.

### Bivalve C3-Like-a Subunit Induces an Inflammatory Response

The consequences of high circulating levels of ScoC3-like-a in razor clams was assessed by measuring a candidate gene of the inflammatory response (*NF-κB*) and a proinflammatory cytokine (*TNF-α*) *in vivo*. Injection of Sco-C3-like-1a significantly up-regulated (p < 0.001) *ScNF-κB* in the liver 4 and 8 h post injection and *ScTNF-α*, 1, 2 and 4 h post injection (**Figures 5C, D**). Injection of ScoC3-like-2a significantly up-regulated (p < 0.001) *ScNF-κB*, 1, 2, 4 and 8 h post injection and *ScTNF-α* at 4 and 8 h post injection. ScoC3-like-3a injections provoked a significant up-regulation (p < 0.001) of *ScNF-κB* and *ScTNF-α* at 1, 2, 4 and 8 h post injection (**Figures 5C, D**).

## Discussion

**Figure 6:**
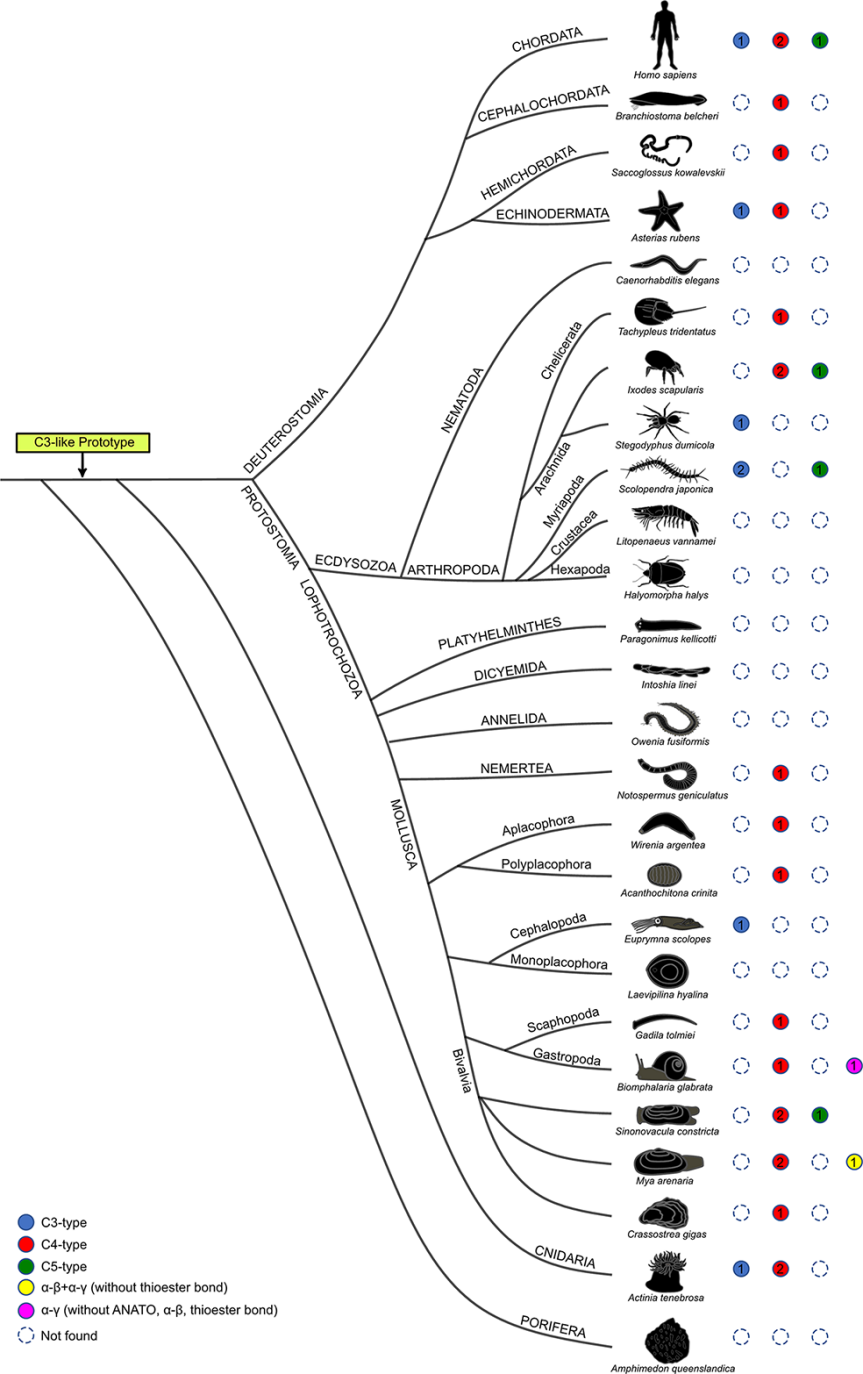
Structural homology of the predicted non-vertebrate C3-like proteins with the vertebrate C3, C4 and C5 proteins. Circles of different color represent the distinct structural isoforms predicted. Human C3, C4 and C5 are represented at the top for comparison. Dashed circles represent gene absence and the numbers inside the colored circles indicate the number of proteins with similar structure found in the species. Blue circles, indicate that a C3-like protein is predicted with a functional structure consistent with the vertebrate C3 (which contains the α-β cleavage site, ANATO and thioester bond); Red circles, indicate that a C3-like isoform is predicted with a functional structure similar to vertebrate C4 (α-β and α-γ cleavage sites, ANATO and thioester bond); Green circles, indicate that a C3-like isoform is predicted with a functional structure similar to vertebrate C5 (α-β cleavage site and ANATO). Two other C3-like protein structures (colored yellow and pink) that have no representatives in vertebrates were identified in Mollusca.

The phylogenetic analysis in the present study indicates the appearance of multiple *C3-like* genes in cnidaria, protostomes and invertebrate deuterostomes was independent of the process that gave rise to the *C3, C4* and *C5* genes in vertebrates. In phyla where innate immunity is the main defense mechanism appearance of multiple C3-like genes is the prevalent evolutionary model. A putative *C3-like* prototype gene emerged early in evolution after Porifera and during the species radiation it was lost from some species genomes while in others, lineage and species-specific gene duplications occurred which probably contributed to structural and functional diversity (**Figure 6**). In the bivalve mollusk, the Chinese razor clam, three paralogue *C3-like* genes emerged by species-specific gene duplication and shared functions with the vertebrate orthologues.

The complexity of mapping C3 evolution is associated with the greater importance of protein domains and protein structure for function and family member assignment than the primary amino acid sequence. The elucidation of the crystalline structure of mammalian C3 (3), which uncovered high domain/structural similarities with C4 and C5 despite substantial primary sequence divergence consolidated this concept (10). This may explain why evolutionary models based on the comparison of the primary amino acid sequence of non-vertebrate *C3-like* genes fail to cluster gene isoforms within phyla and generate ambiguous evolutionary models. Nonetheless, as observed for vertebrate C3, C4 and C5 high domain/structural similarities existed between the deduced proteins of different *C3-like* gene isoforms (**Figure 6**).

Common structural features of vertebrate C3 and C4 were identified in C3-like isoforms including in the Cnidaria phylum where it first emerged (9, 27, 39). This suggests that the ancestral gene already contained the structural elements responsible for the modern proteins functions and explains the structure/function conservation between vertebrate C3, C4 and C5 and cnidarian, protostome and invertebrate deuterostomes C3-like despite their evolutionary distance (**Figure 6** **)**. In the case of the Chinese razor clam, duplication led to divergence of C3-like isoforms with secondary loss of some structural domains in ScoC3-like-3 and associated functional modifications, which echoes what occurred to vertebrate C4 and C5. The characteristic three-chain structure, α-β cleavage site and α-γ cleavage site of vertebrate C4 was identified in ScoC3-like-1 and ScoC3-like-2 deduced proteins and the two-chain structure, α-β cleavage site and absence of the α-γ cleavage site characteristic of C5 (2, 15) was identified in ScoC3-like-3. In other Mollusks two other C3-like structural isoforms, specific to this phylum, were also predicted. These were found in several gastropods and the deduced proteins had lost important functional domains including the ANATO domain that is responsible for the function of the protein a-subunit raising question about their function (**Figure 6**).

With few exceptions (Gastropods and Scolopendromorpha) a stable and conserved multi-chain structure is the main characteristic of C3-like proteins, which contrasts with the members of the A2M superfamily where no α-β cleavage site exists (11). The structural similarities between deduced C3-like proteins and the TEP superfamily are consistent with its proposed origin from the TEP gene family duplication (1, 9, 40). The *C3-like* gene duplication in Hexacorallia and loss of the α-γ cleavage site in one of the duplicates is reminiscent of the situation that arose in the vertebrate duplication that generated the *C3*, *C4* and *C5* genes (11–15). Although in early vertebrates like the hagfish (*Eptatretus burgeri*), the α-γ cleavage site is still evident in the deduced C3 protein (41, 42) and generates a mature protein with 3 chains. The loss of the α-γ cleavage site in non-vertebrates and vertebrates, was a crucial step in the functional differentiation of the gene duplicates.

The loss of the α-γ cleavage site in *C3-like* occurred independently many times in cnidaria, protostomes and invertebrate deuterostomes during lineage or species-specific gene duplications (**Figure 3**). This was clearly exemplified by the genes and their deduced proteins in the razor clam. *ScoC3-like-1* and *ScoC3-like-*2 generated the more ancient three-chain type protein, while *ScoC3-like-3*, lost the α-γ cleavage site and belongs to the two-chain type. Functional characterization of the razor clam C3-like isoforms generating three-chain or two-chain proteins unveiled the activities of the a-protein subunit, which were reminiscent of vertebrate C4 and C3/C5, respectively (43, 44). Furthermore, the *C3* gene (encoding a two-chain protein) in advanced vertebrates has a specific functional role in the complement signaling pathway, however the *C3* gene of cyclostomes (encoding a three-chain protein) still exhibits a broad activity spectrum (45–47). The functional studies of C3-like isoforms and C3, C4 and C5 in vertebrates fully support the notion that the loss of the α-γ cleavage site contributed to functional differentiation of C3, C4 and C5 family genes (48–50).

From a functional perspective the thioester bond directly interacts with the surface of foreign targets in vertebrate C3 and C4 but not C5 (5, 40) and C3-like in the horseshoe crab (Crustacean, Arthropoda phylum) that retains the thioester bond also bind to the surface of bacteria (30, 39). In vertebrate C3 the thioester bond and the two conserved amino acid residues (His and Glu) in proximity guide foreign target binding and the lack of a thioester bond in C5 explains its functional divergence (5, 40). The results of the phylogenetic and sequence analysis revealed that secondary loss of the thioester bond in C3-like occurred independently many times in cnidarian, protostomes and invertebrate deuterostomes during lineage or species-specific gene duplications. The consequences of *C3-lik*e gene duplication on the function of the complement system in Mollusca have not previously been described and infrequent reports of *C3-like* genes and immunological activity exist in protostomes (17, 18, 30, 39, 51). The functional significance of the structural changes in the non-vertebrate C3-like proteins exemplified by the results of the razor clam is reported for the first time. Based on structure/function relationships the razor clam genes were divided into 2 groups (ScoC3-like1/2 and ScoC3-like-3) that mirrored the modified protein structure found in vertebrate C4 and C5 (**Figure 6**) and both ScoC3-like-1 and 2 were potent hemolytic factors that bind to the surface of pathogens (14, 33). In vertebrates, even though C5 lost the thioester bond, C5b is part of the MAC complex that causes cell lysis. ScoC3-like-3b also lacks the thioester bond but is not a hemolytic factor and its function in the mollusca complement pathway remains to be established.

The complement a-protein subunit is produced from the ANATO domain, which contains six conserved cysteine residues and one cleavage site (35). The convertase cleavage site of the ANATO region (L-R) was conserved across most vertebrates (35, 44) and was missing from the sea cucumber (*A. japonicus*, R-R) (20), Ciona (*C. intestinalis*, Q-G-R) (43) and sea squirt (*Halocynthia roretzi*, T-S-R) (52). Nonetheless, although the L-R site was missing in cnidaria, protostomes and invertebrate deuterostomes LC-MS of razor clam C3-like revealed cleavage occurred at non-consensus sites (e.g V-N-R, R-N-R) to generate the a and b-protein subunit. The anaphylactic activity of the C3-like-a protein subunits in razor clam differed in potency (ScoC3-like-3a > ScoC3-like-1a, ScoC3-like-3a > ScoC3-like-2a) much like the human C3/C4/C5-a protein subunit (35, 53). The chemotactic activity of the razor clam C3-like-a protein subunits on hemocytes and mice macrophage was coherant with their structural and functional conservation with vertebrate C5a activity on neutrophils, monocytes, and macrophages (54) and C3a on eosinophils and mast cells (54, 56, 57). A C3-like-a protein subunit that induces chemotaxis of ascidian (*Pyura stolonifera*) hemocytes was proposed to act *via* a C3aR-dependent mechanism due to the conserved high-level structure of ascidian C3-like-a and vertebrate C3a (58). The recent identification of a complement a-subunit receptor protein in the Heterodonta clade (29) leads us to speculate that the mechanisms determining ScoC3-like-3a induced chemotaxis of bivalve hemocytes may also be *via* a C3aR-like dependent pathway.

## Conclusion

An evolutionary model for the C3 gene family in metazoans was developed and revealed gene family expansion occurred by both lineage and species-specific duplications in cnidarian, protostomes and invertebrate deuterostomes. The main structural domains characteristic of vertebrate C3, C4 and C5 were already present in the ancestral genes and differentiated independently during the evolution of *C3-like* genes. Conservation of the ancestral prototype C3 protein domains in cnidarian, protostomes, invertebrate deuterostomes and vertebrates were associated with functional homology and indicates parallel evolution occurred. The complement system provides an example of how proteins with a strong link between domain structure and function influence gene evolution.

We demonstrate that the parallel functional evolution of the C3, C4, C5 and C3-like genes resulted from a common process, the secondary loss of specific functional domains due to gene duplication, which corroborates the *secondary loss evolutionary model* of Nonaka and Kimura (10).

The detailed evolutionary and functional analysis of C3-like in bivalves leads us to propose that overall, the complement system in mollusks shares the core functions of C3, C4 and C5 in vertebrates. The results of the present study, taken with data from other studies (14, 28, 33, 34) indicate the complement system in cnidarian, protostomes and invertebrate deuterostomes acquired unique characteristics: 1) it does not rely on a MAC structure to cause lysis and 2) novel reaction cascade pathways are triggered by C3-like.

## Materials and Methods

### Animals, Experimental Conditions, and Tissue Sampling

Chinese razor clam (*Sinonovacula constricta*) is a non-endangered species. Adult razor clams (body weight 8.5 ± 0.5 g, length 5.0 ± 0.3 cm) were collected from Donghang Farm (Zhejiang Province, China) and used in the experiments. Approximately 300 adult razor clams were maintained in a 40 L tank (80×50×30cm) with oxygenated freshwater at 24 – 25°C and 20 ‰ salinity. Half of the tank water was renewed daily. Experimental animals were anesthetized on ice before tissue collection and were opened by cutting the adductor mussel and hinge with a blade. The SCH was collected from the cardiocoelom using a sterile 1000 μL syringe (14) and each sample represents a pools of 9 animals. The SCH was centrifuged to separate the SCHC and filtered twice (0.22 μm) to remove any cells, bacteria, or other debris. The collected SCH was used in: Western blot (WB), immunoprecipitation (IP), hemolysis assays and mass spectrometry (MS). The pelleted SCHCs were washed and resuspended in Tris Buffered Saline (TBS, 50 mM; Tris-Cl pH 8.0; 300 mM NaCl) before determination of cell migration and phagocytic activity. All tissues collected were immediately frozen in Liquid nitrogen and stored at -80°C.

### Database Searches, Phylogeny Analysis and Protein Motif Annotation

Human C3, C4 and C5 sequences were used as queries to retrieve putative C3-like genes/transcripts (*e-value* ≤ 1e ^-40^) from 66 species representative of different animal phyla (Anthozoa, Nemertea, Mollusca, Arthropoda, Echinodermata, Chordata) in public databases (**Supplementary Table 1**). The identity of retrieved sequences was confirmed by searching against NCBI (nr, taxid:9606). Multiple sequence alignments (MSA) were made using the MUSCLE algorithm (59) and the deduced amino acid sequence (60). For the phylogenetic analysis removal of gaps and improvement of alignments was achieved by manual editing of the MSA and two tree-building methods were used: Maximum Likelihood (ML) and Bayesian Inference (BI). The BI tree was built in the CIPRES Science Gateway v3 using a WAG substitution model (selected using model test-ng 0.1.5) and run on XSEDE v3.2.7a with 1.000.000 generation sampling and probability values to support tree branching. The ML tree was built in PhyML 3.0 from the ATGC bioinformatics platform (http://www.atgc-montpellier.fr/phyml/ with the same model and 100 bootstrap replicates. Trees were displayed in FigTree 1.4.3 (http://tree.bio.ed.ac.uk/software/figtree), rooted with human CD109 and A2MG and edited in the Inkscape program (https://inkscape.org). An MSA of the full length deduced amino acid sequence of vertebrate C3, C4 and C5 and non-vertebrate C3-like was used to identify common protein domain structures according to (3, 36, 37) and specific motifs/domains that included the α-β cleavage site, ANATO domain, the α-γ cleavage site, Thioester bond site and the downstream His-Glu amino acid residues were analyzed in detail. MSA were displayed using TBtools (38) software.

### Mass Spectroscopy

Mass spectroscopy (MS) was used to determine if ScoC3-like-3 was cleaved and if cleavage liberated C3-like3a and C3-like-3b protein subunits. Complement activation reactions were carried out by adding LPS (0.2 μg) to SCH (200 μL) and incubating for 1h at room temperature. The reaction mix (20 μL of the reaction mix) was analyzed on a 10% SDS-PAGE polyacrylamide gel stained with Coomassie Brilliant Blue. The region of the gel between 7-15 kDa (corresponding to the predicted size of C3-like-3a) and between 60-135 kDa (corresponding to the predicted size of C3-like-3b) were excised and analyzed by MS (UHPLC Systems, UltiMate 3000, Thermo Fisher, USA) and the output data analyzed using Mascot 2.2 software (see **Supplementary Methods**).

### Hemolytic Activity

The hemolytic activity of three ScoC3-like isoforms was determined using rabbit erythrocytes in TBS and protein specific antisera (ScoC3-like-1b and ScoC3-like-2b (34) and ScoC3-like-3b antisera). For the hemolysis assays, triplicate 200 μL reactions were set-up for each treatment and incubated at 28°C with occasional mixing for 5 h (**Supplementary Table 4**, see **Supplementary Methods**). The experiment was repeated on two other independent occasions. The hemolysis rate was calculated using the formula: (Sample OD − PBS OD) × 100/(water OD − PBS OD). A hemolysis rate of less than 5% was taken to indicate no hemolysis occurred [Mayer (61)].

### Cell Chemotaxis Assay

The capacity of each isoform of ScoC3-like-a (0, 5, 10, 20 and 40 μg/ml) to stimulate chemotaxis was tested with SCHC and with a mammalian monocyte macrophage cell line (J774A.1) derived from tumors of BALB/c mice (FH0329, FuHeng Cell Center, Shanghai, China). Three independent assays were performed. Cells were activated by exposure for 12 h to 200 nm/ml LPS (from *Escherichia coli* O111:B4, Sigma-Aldrich, USA) and Transwell chemotaxis assays were performed at 28°C for 3 h with the SCHC cells and at 37°C for 2 h with the J774A.1 cells (see **Supplementary Methods**). Cells that migrated to the lower chamber were analyzed with a flow cytometer (BD C6Plus, BD Biosciences, USA). Control assays included the use of C3-like-a proteins exposed to heat-treated, trypsin-treated, or heat inactivated trypsin. A μ-Slide Chemotaxis assay (μ-Slide Chemotaxis ibiTreat, Ibidi, Germany) (62, 63), was used to confirm the observed effects of ScoC3-like-3a (see **Supplementary Methods**). The μ-Slide was observed using an inverted microscope (DMI8, Leica, Germany) and the cell migration captured by photographing preparations every 2 min over 90 min. Cell trajectories (32 and 34 cells in C3-like-a exposed and BSA group, respectively) of the three assays were analyzed using the ImageJ (NIH, Bethesda, MD) program and the Ibidi chemotaxis with the migration tool. Displacement of center of mass (COM) and the forward migration index (FMI) was determined (64).

### Phagocytosis Assay

Phagocytosis by SCHC of heat killed bacteria (*S. aureus* and *V. anguillarum*) labelled with FITC was assessed in the presence and absence of the recombinant ScoC3-like-a proteins by flow cytometry (BD C6Plus) and dot plots collected (see **Supplementary Material**). Three independent assays were set up. The phagocytic ratio of SCHC was established by applying the formula: 100% × (total SCHC – non-phagocytic SCHC)/total SCHC.

### *In Vivo* Injection of ScoC3-Like-a Protein Subunit

The consequences of high circulating levels of each of the 3 isoforms of ScoC3-like-a subunits *in vivo* was analyzed. Approximately 175 adult razor clams acclimated to the experimental conditions (outlined above) were used and divided into 5 groups (35 animals per group). The control group was injected with 100 μL TBS into the foot and four treatment groups were injected with either 40 μg of BSA or the recombinant ScoC3-like-1a, ScoC3-like-2a, or ScoC3-like-3a (see **Supplementary Methods** for recombinant protein production). After injection six individuals from each group were killed at 0, 1, 2, 4 and 8 h and the liver was dissected out and stored at -80°C until analysis. The candidate genes chosen to monitor the immune response were *TNF-α* and *NF-κB* obtained from a cDNA library (65) (**Supplementary Table 5**). Total RNA, cDNA and expression analysis were performed as described above.

## Data Analysis

SPSS 19.0 statistical software was used for data analysis. Data from qRT-PCR and functional assays were analyzed using a Fisher LSD one-way ANOVA to determines significant differences between groups (*p* < 0.05). The data in **Figures 4F**, **G** were analyzed using a Student *t*-test to determine the existence of significant differences between groups (*p* < 0.05). Data are presented as the mean ± SEM. Figures were plotted using GraphPad Prism software (V8.0.2, USA).

## Data Availability Statement

The datasets presented in this study can be found in online repositories. The names of the repository/repositories and accession number(s) can be found in the article/**Supplementary Material**.

## Author Contributions

JL, DN, XL, ZD, and DP planned and supervised the study. MP and JC performed the bioinformatic and comparative analysis. MP and ZL performed the experimental work. MP, JC, and DP analyzed the results and wrote the manuscript. All authors contributed to the article and approved the submitted version.

## Funding

This work was supported by a National Key R & D plan “Blue Granary Science and Technology Innovation” special project (grant number 2019YFD0900700), the National Natural Science Foundation of China (grant number 31472278), the National Natural Science Foundation of China (grant number 32072975) and by the Portuguese Foundation for Science and Technology (FCT) through project UIDB/04326/2020 and from CRESC Algarve 2020 and COMPETE 2020 through the project EMBRC.PT ALG-01-0145-FEDER-022121. ZL was supported by a PhD scholarship from the China Scholarship Council.

## Conflict of Interest

The authors declare that the research was conducted in the absence of any commercial or financial relationships that could be construed as a potential conflict of interest.

## Publisher’s Note

All claims expressed in this article are solely those of the authors and do not necessarily represent those of their affiliated organizations, or those of the publisher, the editors and the reviewers. Any product that may be evaluated in this article, or claim that may be made by its manufacturer, is not guaranteed or endorsed by the publisher.
