# Supplementary material for: Domain-Dependent Evolution Explains Functional Homology of Protostome and Deuterostome Complement C3-Like Proteins
Source: Front Immunol. 2022 Mar 10;13:840861. doi: 10.3389/fimmu.2022.840861 (PMC8960428; doi:10.3389/fimmu.2022.840861)
Supplement: Supplementary file 1 [file DataSheet_1.docx]

**Domain-dependent evolution explains functional homology of protostome and deuterostome complement C3-like proteins**

Maoxiao Peng^1,2,†^, Zhi Li^1,2,†^, João C.R. Cardoso^2^, Donghong Niu^1,3,4^, Xiaojun Liu^5^, Zhiguo Dong^4^, Jiale Li^1,3,4,^*, Deborah M. Power ^2,6,^*

^1^ Key Laboratory of Exploration and Utilization of Aquatic Genetic Resources, Ministry of Education, Shanghai Ocean University, Shanghai, China, ^2^ Comparative Endocrinology and Integrative Biology, Centre of Marine Sciences, Universidade do Algarve, Faro, Portugal, ^3^ Shanghai Engineering Research Center of Aquaculture, Shanghai Ocean University, Shanghai, 201306, China, ^4^ Co-Innovation Center of Jiangsu Marine Bio-industry Technology, Jiangsu Ocean University, Lianyungang, China, ^5^ Department of Biotechnology and Biomedicine, Yangtze Delta Region Institute of Tsinghua University, Jiaxing, Zhejiang, 314000, China, ^6^ Shanghai Ocean University International Center for Marine Studies, Shanghai, 201306, China.

**Supplementary methods and Supplementary Tables (1 to 5)**

**Supplementary methods**

*RNA extraction and cDNA synthesis*

For tissue distribution liver, gills, foot, hemolymph, mantle, gonad, and siphon tissues of nine adult clams were sampled and total RNA (tRNA) was extracted using a RNeasy Plus kit (Qiagen, CA) following the manufacturer’s instructions. Total RNA (tRNA) from three individuals was pooled together in equal amounts to give 1 μg tRNA used for cDNA synthesis (n = 3 pools/tissue). cDNA was prepared using a PrimeScript™ reagent kit with Oligo-dt (25 pmol) and gDNA Eraser to remove contaminating genomic DNA (Takara, Japan) as previously described (14).

*Quantitative PCR*

Transcript abundance was determined using quantitative PCR (qRT-PCR) with specific primers (**Supplementary Table 5**) and a CFX Real Time PCR Detection System (BioRad, USA). Control reactions included a qRT-PCR control and a -RT control. The qRT-PCR reaction efficiencies and r^2^ (coefficient of determination) were all > 90 % and *S. constricta* *18S* rRNA, which did not vary between samples was used as the reference gene. The qRT-PCR reactions were established for a 20 µl final reaction volume containing 400 nM of each primer (2 µL), 6 μL nuclease-free water, 10 μL of 2 × SYBR Premix Ex *Taq*™ (Takara, Japan), 2 µL of the template cDNA (20 ng for the target gene, 5 pg for *18s*). Reactions were performed in duplicate (< 5% variation between replicates) and thermocycling conditions were one cycle of 95 °C for 30 s; 35 cycles of 95 °C for 5 s, 60 °C for 30 s and 72°C for 1 min; and one cycle of 72 °C for 3 min. Melting curve analysis was performed to detect non-specific amplification products and primer dimers.

*Production of recombinant proteins*

Recombinant proteins corresponding to the b-protein subunit of ScoC3-like-3 and to the a-protein subunit of the three ScoC3-like isoforms were produced in a prokaryote expression system. The recombinant ScoC3-like-3b protein was used to generate a polyclonal antiserum in rabbit against the ScoC3-like-3b protein subunit for the hemolysis assay. This protein was generated using the nucleotide sequence corresponding to ^290^F-A^530^ of the deduced protein sequence and was amplified by RT-PCR using specific primers, ScoC3-like-3-F1 and ScoC3-like-3-R1 (200 nM) with a 5’ region compatible with the cloning site of the pET32a vector (Novagen, Germany). The amplicon was sequenced to confirm its identity and then cloned into the *BamHⅠ* and *XhoⅠ* digested pET32a vector. The recombinant proteins corresponding to the ScoC3-like-a protein subunits were used for the chemotaxis, phagocytosis and overexpression assay. In brief, the nucleotide base sequence of ScoC3-like-1a*,* ScoC3-like-2a, ScoC3-like-3a (**Supplementary Figure 6**) was synthesized with the inclusion of an N-terminal His-tag sequence (Sangon Biotech, China) and with the *NcoI* and *XhoI* restriction digestion sites for cloning in the *NcoI* and *XhoI* digested pET28a vector (Novagen, Germany). The recombinant constructs were used to transform *E. coli* (BL2 strain) grown in Terrific Broth containing 50 mg/L ampicillin. Protein expression was induced with 0.5 mM IPTG at 16 °C for 10 h. The bacterial pellets containing expressed proteins were collected by centrifugation at 5,000 × *g* for 10 min. All recombinant proteins were purified using an Ni-NTA purification column (HisTrap HP, GE Healthcare, USA) and eluted with NTAU-X (50mM PBS, pH 7.4, 0.5M NaCl, 8M Urea, gradient concentration of Imidazole) (**Supplementary Figure 7**). Several rounds of column purification and elution in 200 mM imidazole (NTAU-200) buffer yielded 1.5 mg of each recombinant protein with > 80% purity.

*Production of a specific polyclonal antiserum for ScoC3-like-3b*

A polyclonal antiserum specific for ScoC3-like-3b (^290^F-A^530^) was custom produced by a commercial company (Youke, Shanghai, China) using the affinity purified recombinant ScoC3-like-3b (^290^F-A^530^) protein. Western blot (WB) analysis was performed using razor clam hemolymph (SCH) to verify the specificity of the antisera. SDS-PAGE gels (10 %) were used to separate the proteins in SCH (10 µL) and transferred to nitrocellulose membrane. A One Step Western blot kit (CWBIO, China) was used following the manufacturer’s instructions. The working dilution of the primary antisera was 1/1000 and the secondary antisera, goat anti rabbit IgG conjugated to horseradish peroxidase (HRP) (CWBIO, China) was used at a dilution of 1/100. An ECL Western Blot Kit (CWBIO, China) was used for signal development following the manufacturer’s instructions and the reaction was detected using an ImageQuant™ LAS 4000 (GE Healthcare BioSciences, USA)

*Detection of ScoC3-like-3b in razor clam hemolymph*

A specific antiserum for ScoC3-like-3b was generated and Pierce classic immunoprecipitation (IP) Kit (Thermo Fisher, USA) was used following the manufacturer’s instructions. For IP reactions, 280 μL SCH and 40 μg ScoC3-like-3b antisera were mixed and incubated for 2 h at 28 °C to obtain SCH - antibody immune complex. The IP reactions were analysed by 10% SDS-PAGE polyacrylamide electrophoresis and for detection of the protein complexes they were stained with Coomassie Brilliant Blue. The treatment groups analysed by SDS-PAGE included: 10 μL SCH sample (after IP) and the following controls, 10 μL ScoC3-like-3b antisera, 10 μL SCH and 10 μL TBS. The IP assay was repeated in three independent experiments.

*Mass spectroscopy*

Mass spectroscopy (MS) was used to confirm ScoC3-like-3 cleavage. The region of the SDS-PAGE gel corresponding to the predicted size of C3-like-a (7-15 kDa) subunit and C3-like-b subunit were placed in separate tubes and then subject to reduction and alkylation treatments. Trypsin was added to the excised polyacrylamide gel (mass ratio 1:50) and incubated at 37 ℃ for 20 h, desalted, lyophilized, and then dissolved in 0.1% formic acid (FA). Trypsin digested samples were analysed by MS using mass-to-mass ratio and collecting 20 fragment scans (MS2 scan) in full scan mode (UHPLC Systems, UltiMate 3000, Thermo Fisher, USA). Mascot 2.2 software was used to analyse the mass spectrum by comparing predicted peptide fragments to an “in-house” transcriptome library (66). Since the boundary of ScoC3-like-3a was not clearly defined the MS fragments of ScoC3-like-3b were used to identify the cleavage site.

*In vivo bacteria immune challenge*

Pathogenic bacteria *Micrococcus lysodeikticus* (a standard challenge for complement activation) (67-68) and *Vibrio parahaemolyticus* were used. The isolated pathogens were supplied by the Aquatic Pathogen Collection Centre of the Ministry of Agriculture, China. The bacterial challenge was performed according to (33). Briefly, 300 razor clams were selected and *M. lysodeikticus* and *V. parahaemolyticus* (1×10^9^ CFU/mL) were injected into the foot (50 μL, n = 70/ treatment). The control group (n = 70) received sterile PBS alone. At 0, 4, 8, 12, 24, 48, and 72 h after the injection animals were killed and the liver and SCHC of nine razor clams from each group collected. Total RNA, cDNA and expression analysis were performed as described above.

*Hemolytic activity*

To activate the complement signalling cascade in the razor clam, LPS (0.2 ug) was added to SCH and incubated for 1h at 28ºC. For the hemolytic activity assay seven experimental treatments were performed (**Supplementary Table 4**). Two hundred μL of rabbit erythrocytes (2 %) was added to each treatment and incubated for 5 h at 28 °C. PBS was used as the negative control and sterile water as the positive control. The mixture was centrifuged at 3,000 g for 10 min and for the determination of the hemolysis rate the supernatant was collected (200 ul) and the absorbance read at 570 nm using a Spectra Max 190 (Molecular Devices, USA) using a 96 well plate.

*Cell chemotaxis assay*

For the Transwell assays, 0.6 ml of DMEM containing BSA (0, 5, 10, 20 and 40 μg/ml) or an equivalent concentration of each of the recombinant proteins, ScoC3-like-1a, ScoC3-like-2a, ScoC3-like-3a (0, 5, 10, 20 and 40 μg/ml) was added to the lower Transwell chamber. Assays with SCHC cells were carried out using a 6.5 mm diameter Transwell insert and a 3.0 μm pore size (Transwell, Corning, USA). Assays with J774A.1 cells were carried out using a 6.5 mm diameter Transwell insert and an 8.0 μm pore size (Transwell, Corning, USA). A 0.1 ml cell suspension in DMEM of razor clam hemocytes (SCHC) cells (4 x 10^7^) or J774A.1 cells (3 x 10^5^) was added to the upper chamber and cell chemotaxis was assessed. Assays were performed at 28 ℃ for 3 h with the SCHC cells and at 37 ℃ for 2 h with the J774A.1 cells and migrating cells determined by harvesting the cells in the lower Transwell chamber and counting them with a flow cytometer (BD C6Plus, BD Biosciences, USA). Control assays to confirm the specificity of the recombinant proteins included the addition to the lower Transwell chamber of 40 μg/ml of ScoC3-like-1a, ScoC3-like-2a or ScoC3-like-3a recombinant proteins either heat-treated, trypsin-treated, or treated with heat inactivated trypsin. ScoC3 had strong chemotaxic activity on J774A.1 cells a second assay was carried out using a μ-Slide Chemotaxis assay (μ-Slide Chemotaxis ibiTreat, Ibidi, Germany)(63-64). In brief, J774A.1 cells were activated by incubation for 12 h with LPS (200 nm/ml). Then 50 μL of the J774A.1 cell suspension (3 x 10^6^ cells/ml) was added to the μ-Slide prepared with a 1.5 mg/ml bovine collagen I gel (20 μl of 10 x MEM, 20 μl of deionized water, 10 μl of NaHCO_3_ and 50 μl of 1 x RPMI 1640 and 100 μl of 150 μl type I bovine collagen, Advanced BioMatrix, USA) and incubated for 45 min (37 ℃, 5% carbon dioxide incubator).

The μ-Slide was observed using an inverted microscope (DMI8, Leica, Germany) and 65 μl of DMEM was injected into the left reservoir, and 65 μl of DMEM and ScoC3-like-3a protein (40 μg/ml) was injected into the right reservoir. To monitor cell migration photographs at 2 min intervals over 90 min were used to determine migration using the Ibidi chemotaxis and migration tool (65). Cell trajectories were set to (*x*; *y*) = 0 at time 0 h, and two values were established for each treatment: the displacement of centre of mass (COM) and the forward migration index (FMI). The COM corresponds to the spatial average of all cell positions (*x* and *y* coordinates). The displacement of COM was calculated as the difference between initial and final COM values. The FMI was calculated in *x* and *y* directions as the final endpoint migration divided by the total migration distance (69).

*Phagocytosis assay*

Heat killed bacteria (*S. aureus* and *V. anguillarum*) were prepared by boiling them for 15 min and then they were suspended in 1mL CB at 1 × 10^9^ CFU/mL with 50 μL FITC solution (10 mg/mL in DMSO, Sigma-Aldrich) and incubated at room temperature in the dark for 1 h. The FITC-labelled bacteria were washed and 1 × 10^7^ suspended in TBS, mixed with 5 × 10^6^ SCHC and incubated for 2 h in the dark at 25 °C with the recombinant ScoC3-like-1a, ScoC3-like-2a or ScoC3-like-3a (20 μg/ml) proteins. Phagocytosis of heat killed bacteria was assessed by flow cytometry (BD C6Plus) and phagocytic SCHC corresponded to those that left the Gate region. The SCHC that remained in the Gate region were classified as non-phagocytic SCHC. Applying the formula: 100% × (total SCHC – non-phagocytic SCHC) / total SCHC yielded the phagocytic ratio.

**References**

67. Zhang H, Song L, Li C, Zhao J, Wang H, Qiu L, Ni D, Zhang Y. (2008) A novel C1q-domain-containing protein from Zhikong scallop *Chlamys farreri* with lipopolysaccharide binding activity. Fish Shellfish Immunol 25:281-289. doi: 10.1016/j.fsi.2008.06.003

68. Jia B, Jin C, Li M (2020) The trypsin-like serine protease domain of *Paralichthys olivaceus* complement factor I regulates complement activation and inhibits bacterial growth. Fish Shellfish Immunol 97:18-26. doi: 10.1016/j.fsi.2019.12.019

69. Zengel P, Nguyen-Hoang A, Schildhammer C, Zantl R, Kahl V, Horn E (2011) μ-Slide Chemotaxis: a new chamber for long-term chemotaxis studies. BMC Cell Biol 12:21. doi: 10.1186/1471-2121-12-21

**Supplementary Table 1.** List of the sources, and accession numbers of the sequences used in this study (See excel file).

**Supplementary Table 2.** Results of the Mass Spectrometry assay.

|  | Identified peptide Sequence | Restrictions on a/b-fragment | Theoretical molecular weight with a charge | Observed molecular weight with a charge | Charge Numbers | IonScore | Expect Value | Isoelectric point (pI) |
| --- | --- | --- | --- | --- | --- | --- | --- | --- |
| ScoC3-like-1 | CRANK | N-terminal | 663.38243 | 663.38233 | 2 | 25.77 | 3.75E-02 | 6.05 |
| ScoC3-like-1 | SLGTVNR | C-terminal | 884.46246 | 884.46087 | 2 | 31.57 | 2.12E-02 | 8.79 |
| ScoC3-like-2 | VCCDLAR | N-terminal | 893.39679 | 893.39751 | 2 | 20.60 | 8.71E-04 | 5.79 |
| ScoC3-like-2 | SLNSVNR | C-terminal | 789.42132 | 789.41903 | 2 | 31.39 | 3.94E-02 | 9.47 |
| ScoC3-like-3 | QSTNCTNR | N-terminal | 861.44247 | 861.44773 | 2 | 21.24 | 7.08E-01 | 5.50 |
| ScoC3-like-3 | YDEIISFVEEK | C-terminal | 1371.66781 | 1371.66386 | 2 | 61.11 | 2.77E-05 | 4.03 |

Note: The identification fragment displayed by ScoC3-like-1 and ScoC3-like-2 comes from the MS analysis of the C3-like-a subunit. The identified fragment of ScoC3-like-3 comes from the MS analysis of the C3-like-b subunit.

**Supplementary Table 3.** Molecular characterization of the Chinese razor clam C3-like transcripts and predicted proteins.

|  | ORF | 5'-UTR | 3'-UTR | Full length | Theo. Mol. Wt. | Theo. Mol. Wt.  a-subunit | Theo. Mol. Wt.  b-subunit | Identities in  ScoC3-like-1 | Identities in  ScoC3-like-2 |
| --- | --- | --- | --- | --- | --- | --- | --- | --- | --- |
| ScoC3-like-1 | 5256bp | 34bp | 1658bp | 1752aa | 197344.04 | 943663.00 | 186370.54 | - | - |
| ScoC3-like-2 | 5226bp | 35bp | 1034bp | 1741aa | 196130.48 | 932569.00 | 185252.06 | 67% aa | - |
| ScoC3-like-3 | 5106bp | 90bp | 761bp | 1701aa | 191331.87 | 106288.20 | 180181.39 | 35% aa | 37% aa |

**Supplementary Table 4.** Experimental set-up of the hemolytic activity assay

|  | Treatments | | | | | | |
| --- | --- | --- | --- | --- | --- | --- | --- |
|  | A | B | C | D | E | F | G |
| SCH (200 μL) | + | + | + | + | + | + | + |
| LPS (0.2 μg) | - | + | + | + | + | + | + |
| Anti-ScoC3-like-1 (9.8 μg) | - | - | - | + | + | - | + |
| Anti-ScoC3-like-2 (9.8 μg) | - | - | + | - | + | - | + |
| Anti-ScoC3-like-3 (10.6 μg) | - | - | + | + | - | + | + |

**Supplementary Table 5.** Sequence of the primers used in this study

| Primer | Sequence (5'-3') | Comment |
| --- | --- | --- |
| ScoC3-like-3-F1 | CGGGATCCTTTAAGATCCGGGGCA | Fragment amplification of ScoC3-like-3 for antibody production |
| ScoC3-like-3-R1 | CCGCTCGAGTGCCAAAATACGTG |  |
| ScoC3-like-3-F2 | GGTTGTCAAGACCTGGGAGT | Real-time-PCR |
| ScoC3-like-3-R2 | ATCAGTGGTCGAACTGTGGA |  |
| ScNF-κB-F | GGCTTGCTGGGTGATTTGAA | Real-time-PCR |
| ScNF-κB-R | TCCTCCTCTGTCTTCTGGGT |  |
| ScTNF-α-F | TGAGTGGGAAAGGAAGTGGT | Real-time-PCR |
| ScTNF-α-R | GTGGGATCCTCCTCATTGCT |  |
| 18S-F | TCGGTTCTATTGCGTTGGTTTT | Real-time-PCR |
| 18S-R | CAGTTGGCATCGTTTATGGTCA |  |
