# Supplementary material for: Domain-Dependent Evolution Explains Functional Homology of Protostome and Deuterostome Complement C3-Like Proteins
Source: Front Immunol. 2022 Mar 10;13:840861. doi: 10.3389/fimmu.2022.840861 (PMC8960428; doi:10.3389/fimmu.2022.840861)
Supplement: Supplementary file 2 [file DataSheet_2.pdf]

Supplementary figure 1A

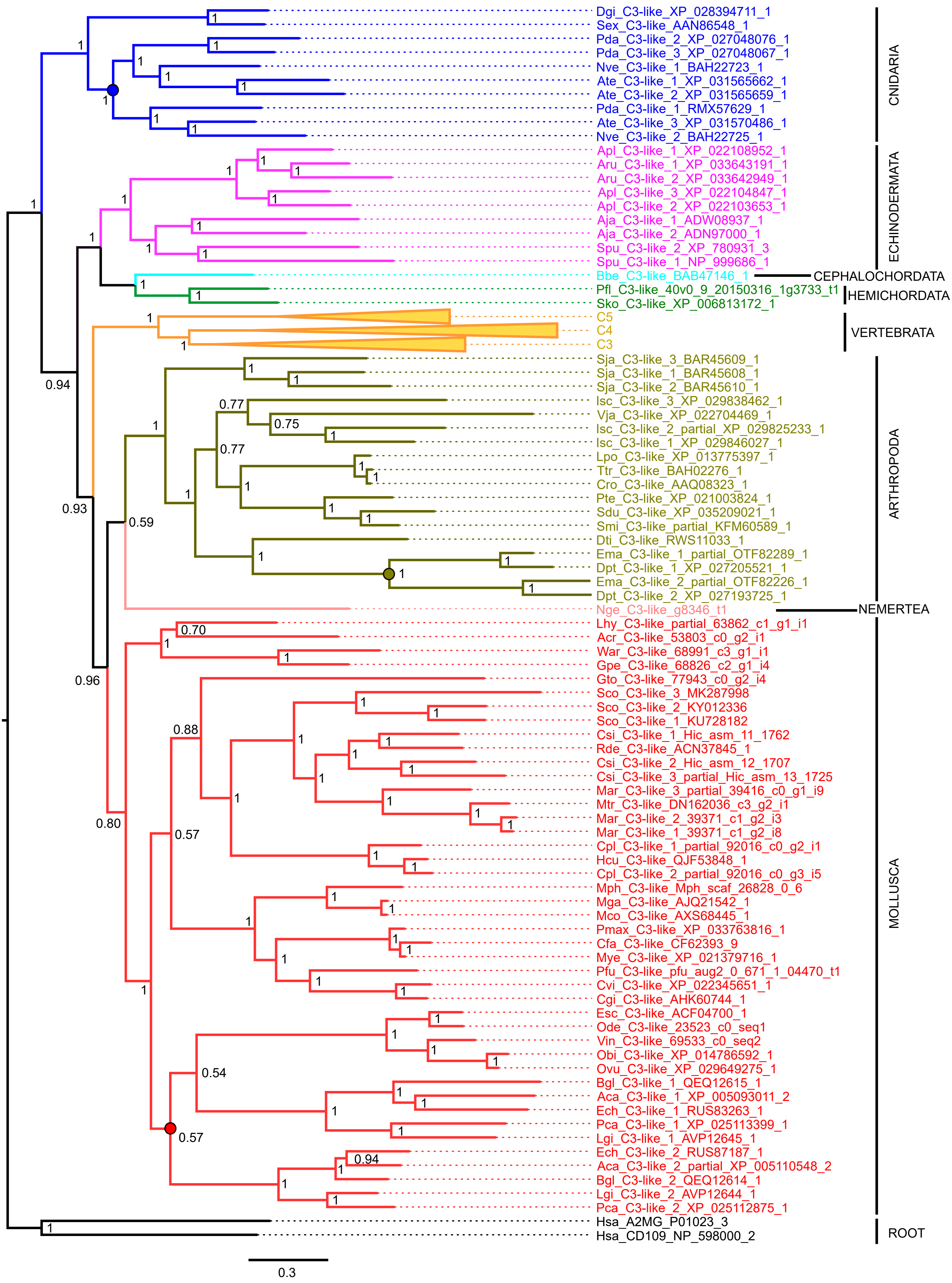

Supplementary figure 1B

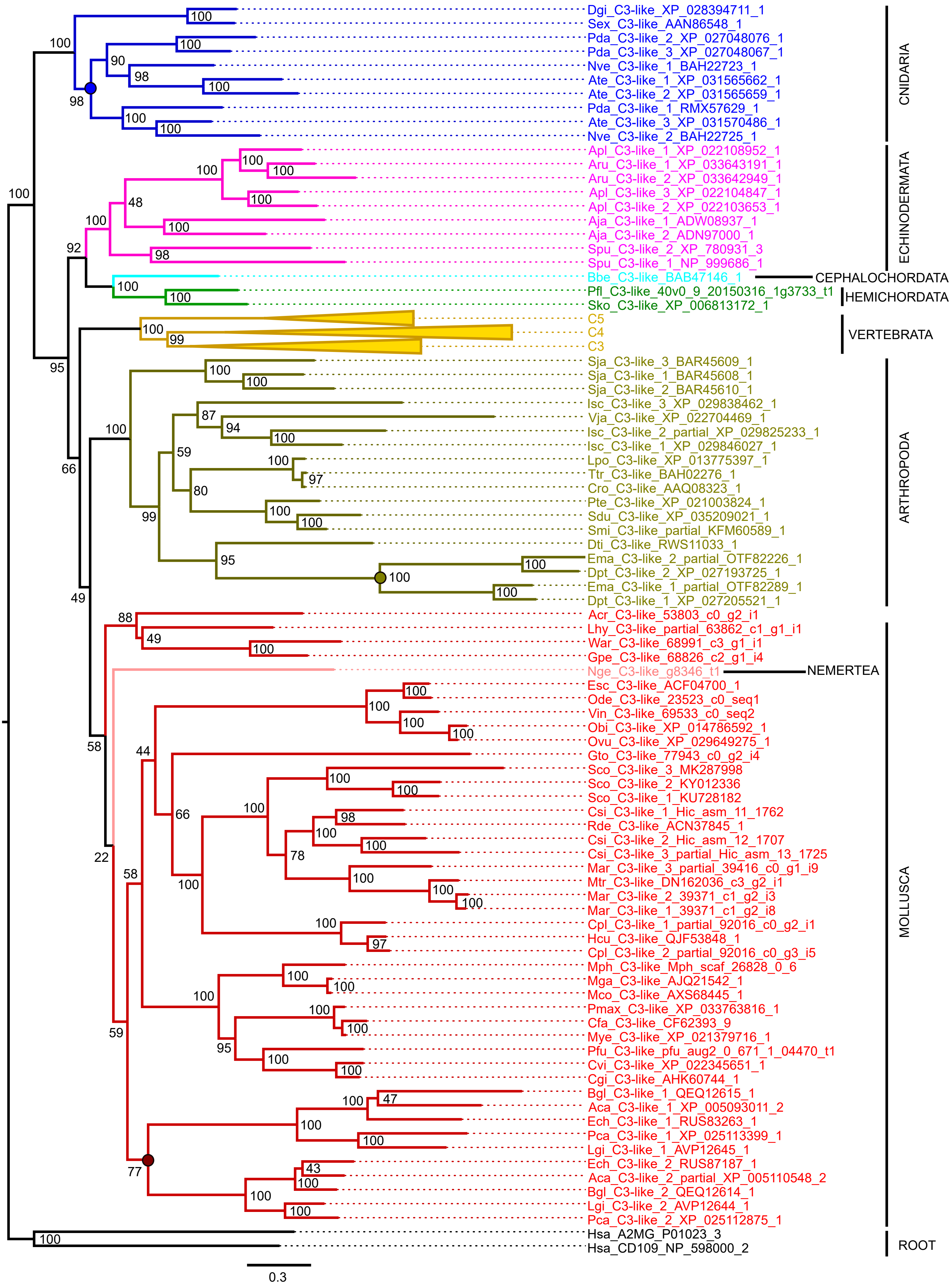

Supplementary Figure 1- Phylogenetic trees of the invertebrate *C3-like* with the vertebrate *C3, C4* and *C5.* A) Bayesian Inference tree with posterior probability values and B) the Maximum Likelihood tree with the bootstrap values. The BI tree was built in MrBayes 3.2 using a WAG substitution model (Aamodel = WAG) with 1,000,000 generations. The ML tree was built in PhyML 3.0 from the ATGC bioinformatics platform (http://www.atgc-montpellier.fr/phyml/) using SMS automatic model selection for the study of protein evolution according to AIC (Akaike Information Criterion). The sources and accession numbers of the sequences used are in **Supplementary Table 1**. Orange highlight and black highlight represent the vertebrates *C3*, *C4* and *C5* clade and the root, respectively. Other colours represent the *C3-like* genes of various invertebrate phylum. The yellow circle represents the two whole genome duplication events that occurred early in the vertebrate radiation and that is proposed to have generated the C3, C4 and C5 family members in vertebrates (13).

Supplementary figure 2A

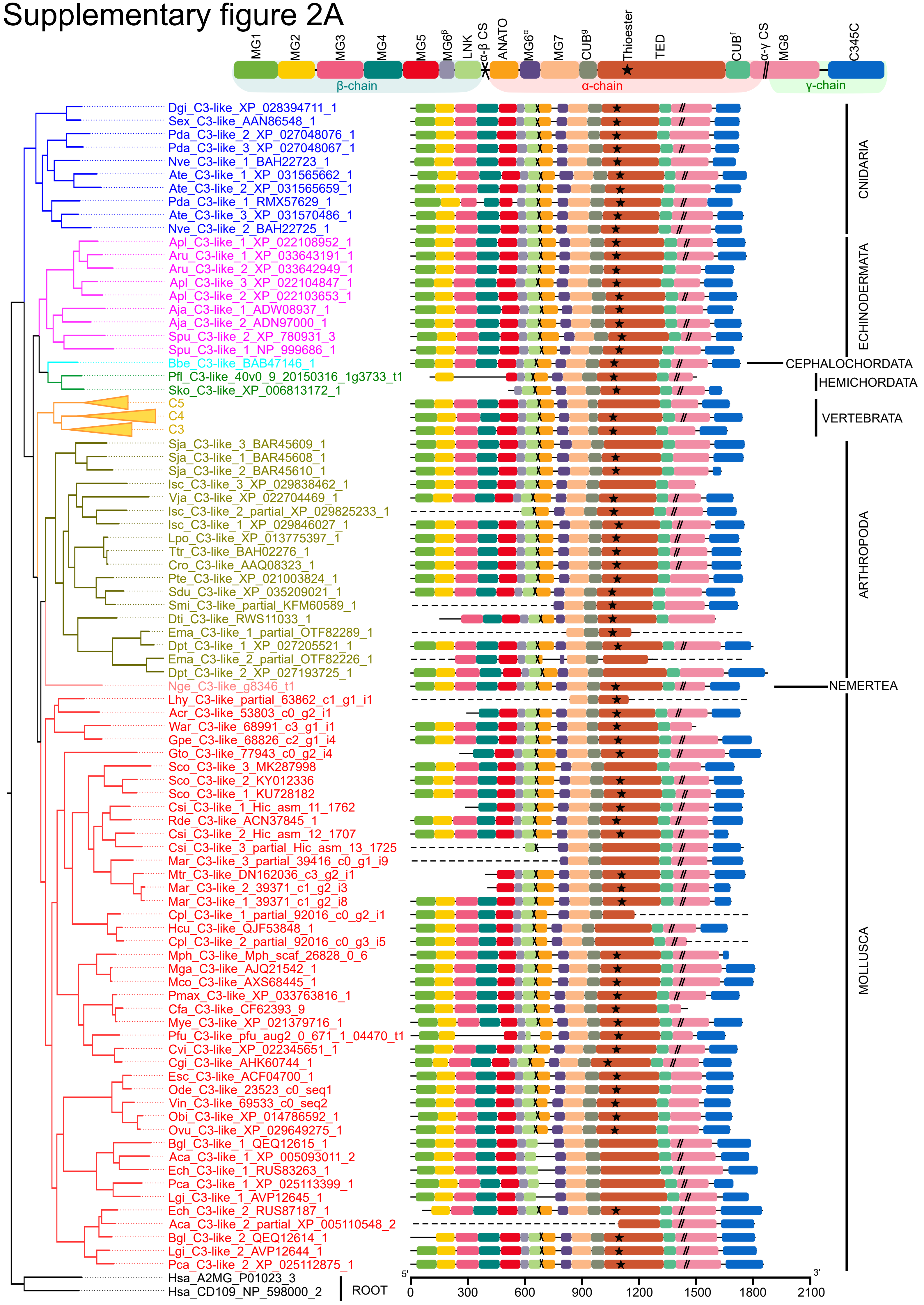

Supplementary figure 2B

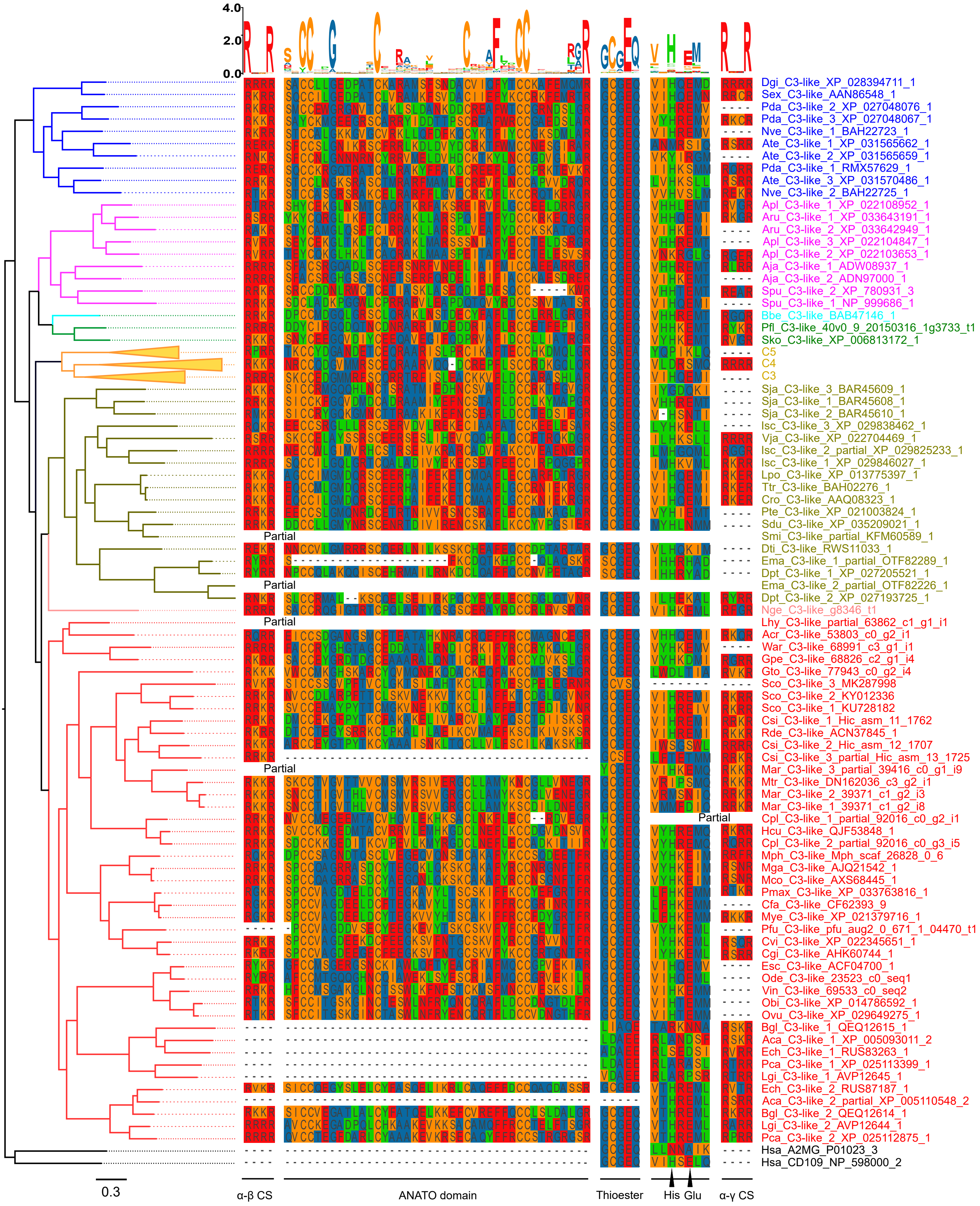

Supplementary Figure 2- Structural and sequence comparisons of the C3-like proteins. A) Amino acid domain alignment of the deduced C3-like proteins. The coloured bars represent the protein domains predicted manually and annotated using the multiple sequence alignment (MSA) based on the predicted domains of human C3, C4 and C5. The cross, five-pointed star and slanted equal sign indicates the α-β cleavage site, thioester bond and α-γ cleavage site, respectively. The dashed line represents incomplete sequences. B) Amino acid sequence alignment of the α-β cleavage site, ANATO region, thioester bond, His-Glu amino acid residues, and α-γ cleavage site. At the top of the alignment letters of different heights indicate amino acid residues with different degrees of conservation, and the bigger the letter the higher the sequence conservation. The ANATO domain contains a relatively high conservation of amino acid residues. The phylogenetic tree represented is a simplified version of **Supplementary Figure 2A**. The sources and accession numbers of the sequences used are in **Supplementary Table 1**.

Supplementary figure 3

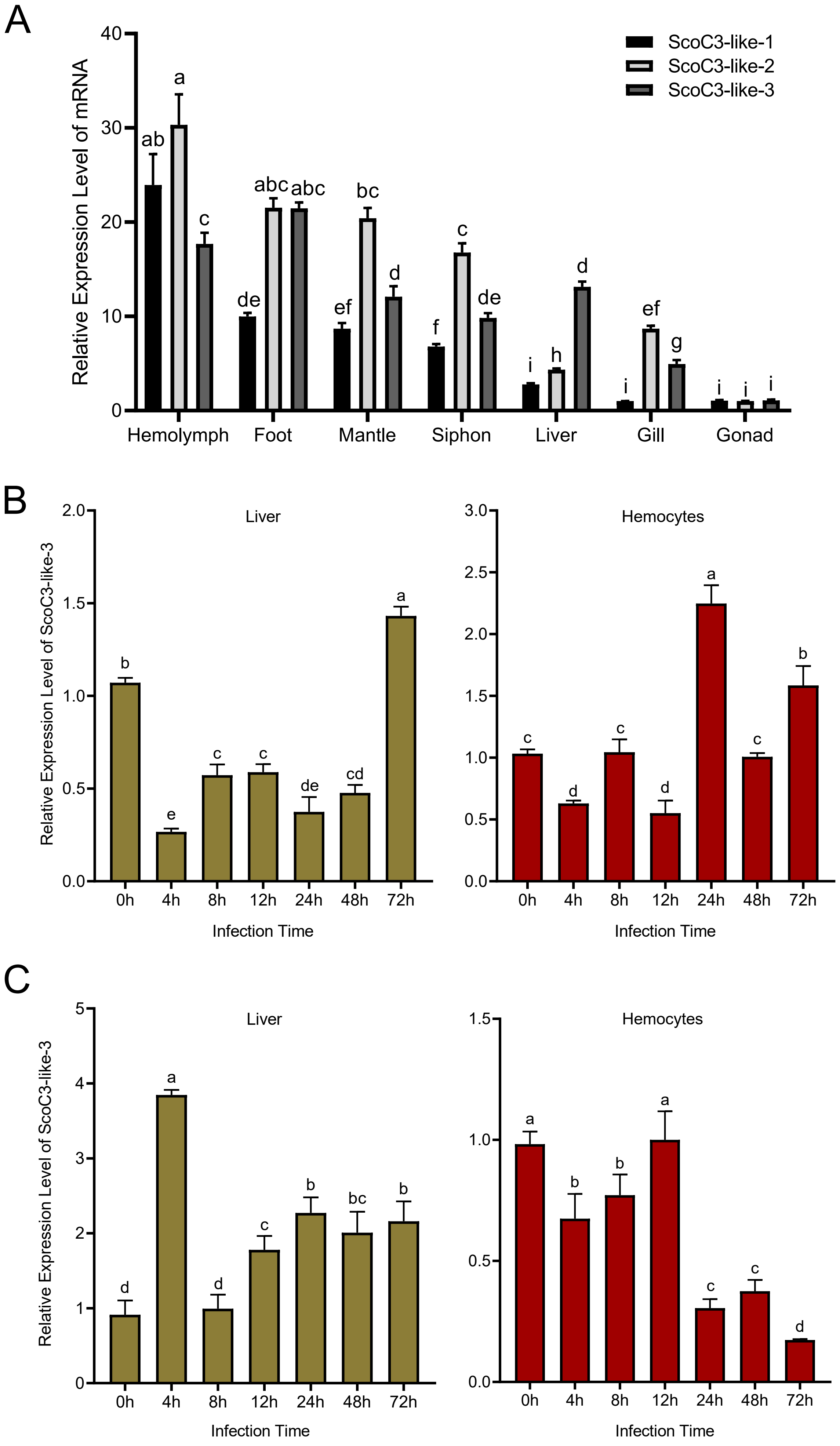

Supplementary Figure 3- Temporal and spatial expression of *ScoC3-like* genes after bacterial challenge. A) Relative expression of the three *ScoC3-like* transcripts in seven healthy tissues from razor clam. Relative expression levels are expressed as fold-change in comparison with expression of *ScoC3-like-1* in the gonad. Data for *ScoC3-like-1* and *ScoC3-like-2* was obtained from previously published studies (14,33). B) The effect of *V. parahaemolyticus* challenge on expression of *ScoC3-like-3* in the liver and hemocytes. Relative expression levels are expressed as fold-change relative to 0h. Significantly increased ($p < 0.001$) *ScoC3-like-3* transcript abundance at 24 h and 72 h in hemocytes and at 72 h in the liver. C) Effect of *M. lysodeikticus* immune challenge on the expression of *ScoC3-like-3* in the liver and hemocytes. Relative expression levels are expressed as the fold-change in comparison to gene expression at 0h. Significant up-regulation ($p < 0.001$) of *ScoC3-like-3* occurred in the liver at 4, 12, 24, 48 and 72 h and significant down-regulation occurred in hemocytes at 24, 48 and 72 h. Bars represent the mean ± SEM ($n = 3$). The different letters above the bars denotes samples that are significantly different ($p < 0.05$).

Supplementary figure 4

A

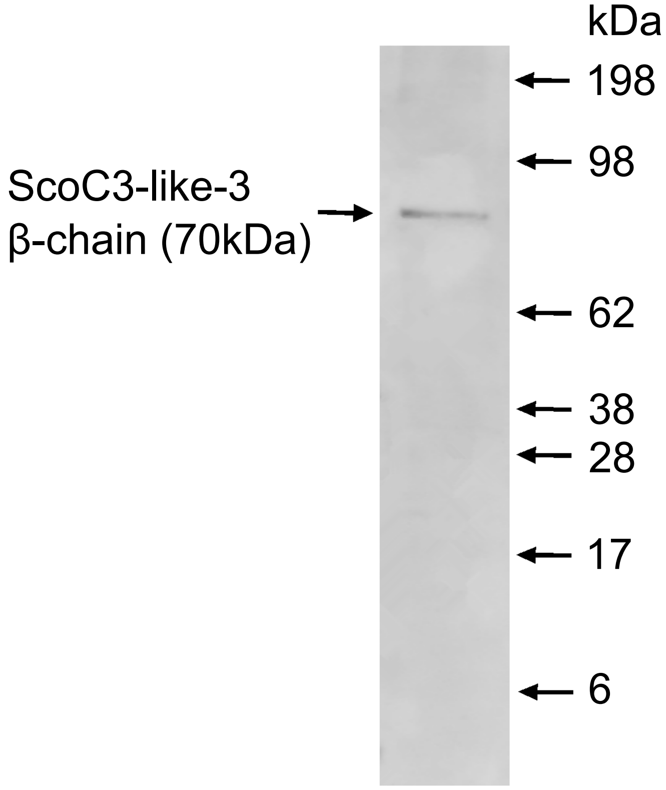

B

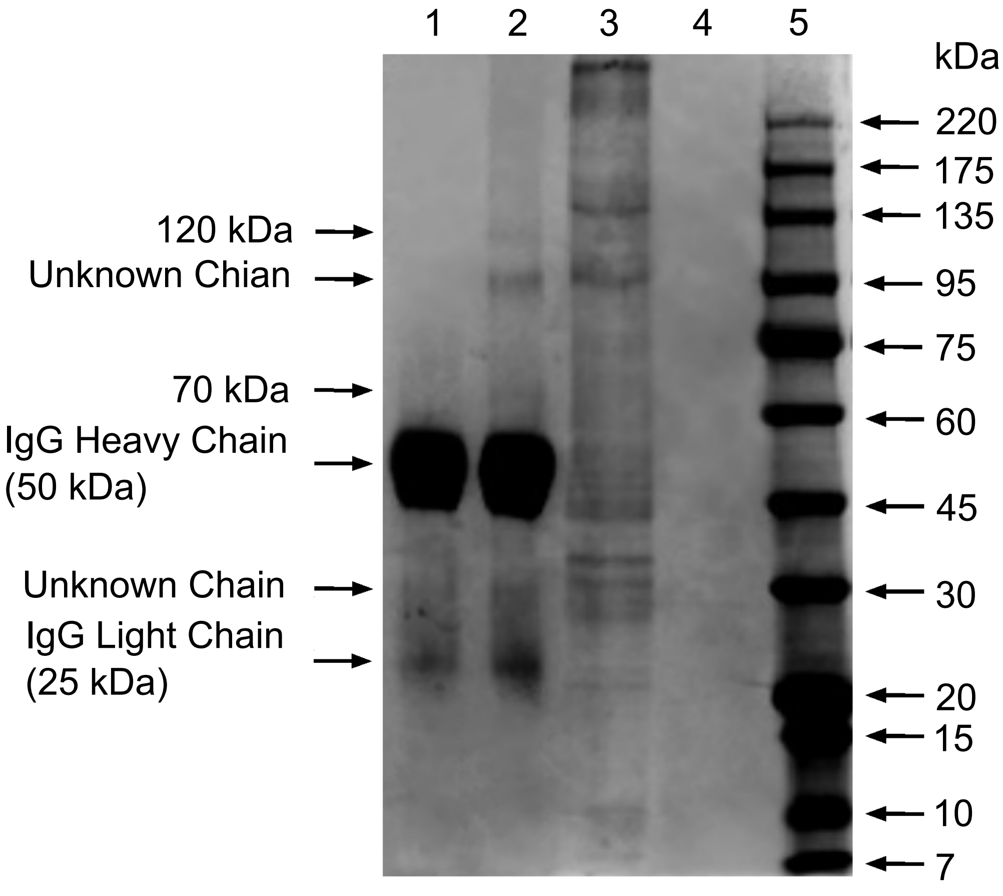

Supplementary Figure 4- Characterization of the Anti-ScoC3-like-3. A) Specificity of Anti-ScoC3-like-3 showing a 70-kDa band similar to the size of the predicted ScoC3-like-3 β-chain in western blot. B) Antibody specificity for ScoC3-like-3. Lanes 1 to 5 represent: ScoC3-like-3 antibody, immunoprecipitation treated sample, SCP treatment at 100°C for 5 min sample, TBS wash buffer and protein ladder, respectively. Six proteins were immunoprecipitated (IP) from the hemolymph by the ScoC3-like-3 antisera. SDS-PAGE of the six IP proteins revealed they had approximate molecular weights of 120, 95, 70, 50, 30 and 25 kDa, respectively. The intensely staining protein bands at 50 and 25 kDa corresponded to the predicted molecular weight of the heavy and light chains of the rabbit polyclonal anti- ScoC3-like-3 IgG. The theoretical molecular weight of ScoC3-like-3 chain is 118.4 kDa and ScoC3-like-3 β chain is 74.4 kDa, which was coherent with the 120 and 70 kDa IP proteins identified by SDS-PAGE. The identity of the IP proteins at 95 and 30 kDa was not established.

Supplementary figure 5A

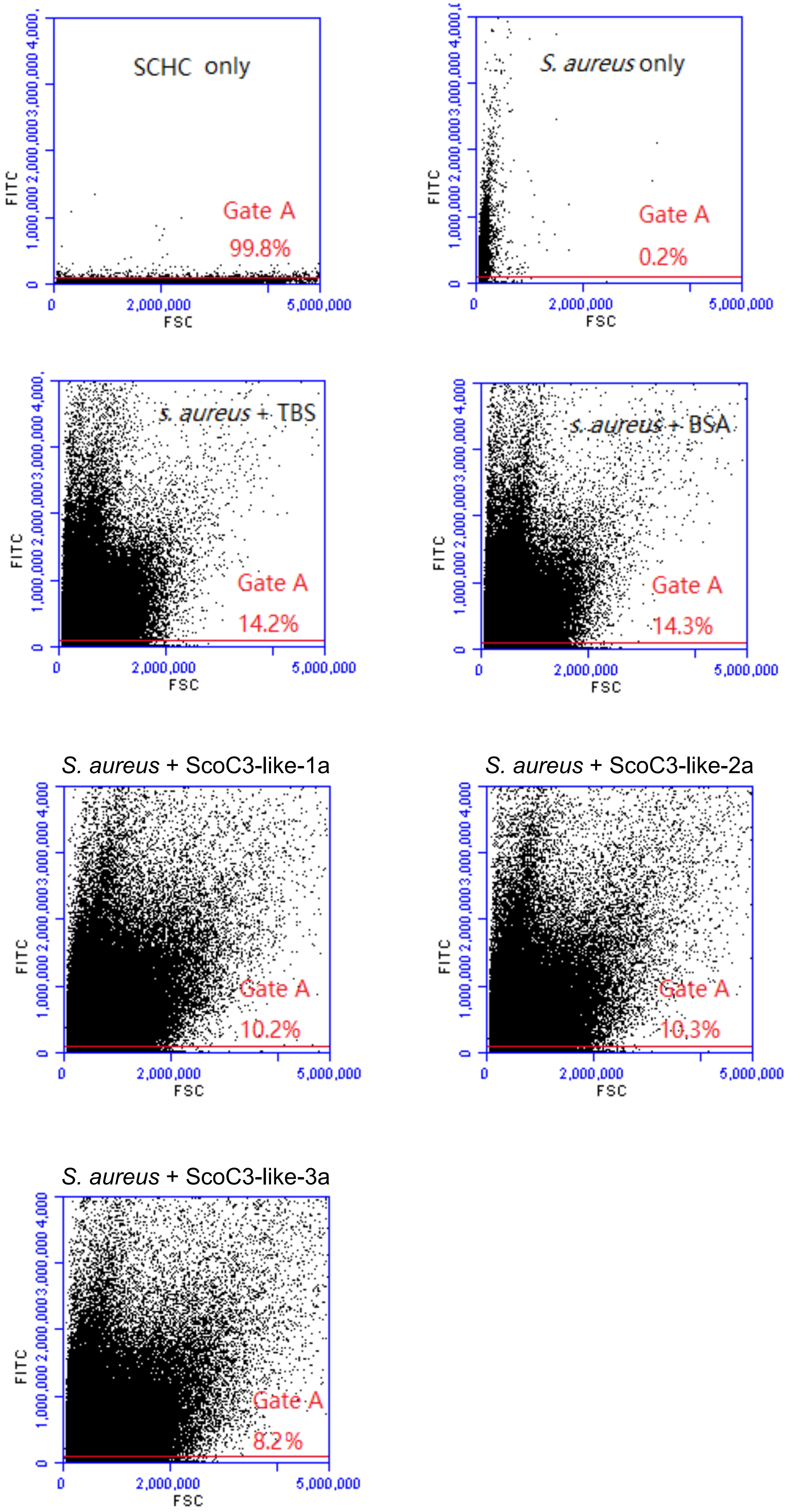

Supplementary figure 5B

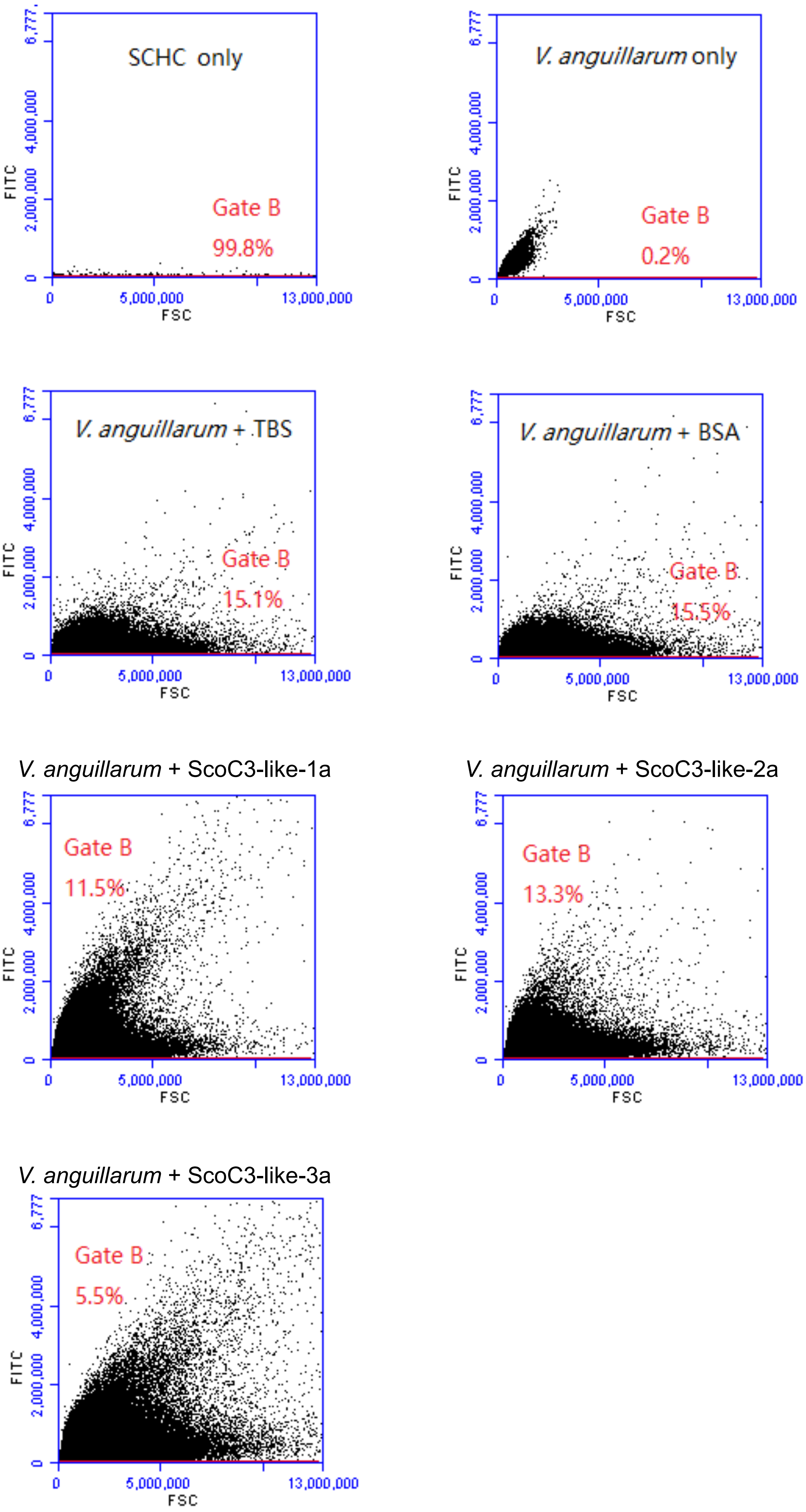

Supplementary Figure 5- Flow cytometry analysis of SCHC in the presence of two S. *aureus* and *V. anguillarum*. Different plots represent the results of primed SCHC with TBS and BSA (controls) and with the three recombinant ScoC3-like-a protein subunits before bacteria exposure A) Plots of SCHC exposed to heat-killed *S. aureus*. Gate A threshold value (percentage) was defined by the dot plots of *S. aureus* only and SCHC only. B) Plots of SCHC exposed to heat-killed *V. anguillarum*. Different plots represent the results of primed SCHC with TBS, BSA and three recombinant ScoC3-like-a protein subunits before bacteria exposure. Gate B was defined by the dot plots of *V. anguillarum* only and SCHC only. In A) and B) the dot plots were selected from one experiment, but three independent experiments were run to confirm the phagocytic activity of SCHC.

Supplementary figure 6

```
                     Nco I     6*HIS tag

ScoC3-like-1a    1   ccatgggcCACCACCACCACCACCACAGCATTGGTAAATGTCGTGCCAACAAGGTTTGCTGTGAAATGGCTTATATATTTACGGAGGACC
ScoC3-like-2a    1   ccatgggcCACCACCACCACCACCACAATACAGACGAATGTCCTGCCGACAAGGTTTGCTGTGATTTGGCTCGAACTTTCGCAAAGAAAC
ScoC3-like-3a    1   ccatgggcCACCACCACCACCACCACAGCGTTGATGATAAAGGATCCTGTAAAACAGAGGCAATATGCTGTAGCAGCGGAGTTGAGTATG

ScoC3-like-1a   91   TTATTTACGATGGCGACGAAATCGAGGATCCGTACACTACATGCATGGGCAAGGTGAATGAGATTAAGGACAAGCAGACTTCGAATGGCG
ScoC3-like-2a   91   AAAATGACACCAAATGGAATGGGGATCCCTTCACTACATGTTTGAGTAAGGTTATGGAAAAGAAAGTCGAACTGACATCAAAGGGTGAAC
ScoC3-like-3a   91   CCAATGAGTATTTTAACGAGAACATTGACAAACAAGACATCGACCCATTCACCGTTTGCCTTGGGAAAACCTCTATTCTTGCCCACGAGA

ScoC3-like-1a  181   AAATGGTTTGGCCAACGAAATGTCTAATCGCCTTCTTTGAGACATGTGTCTATGCGCTGGAGGAATATCTTTCTCCTACAGACACTGAAG
ScoC3-like-2a  181   TAGTTTGGCCTACGAAGTGTCTTATCGCCTTTTTCAAGACCTGTGTTTATACTCTAGAGGAGTCTCTATCTTCGAATGACAATGATGGTC
ScoC3-like-3a  181   AGAAGGAAAACGGAGAGTACGCCTGGCTGACAAAGTGTCTTCTAGCTTTTTATGAGAGCTGCTCAGACCATCTTGATAGTCTCATTGAAC

                                                                  Xho I

ScoC3-like-1a  271   ACATTGGAAAGTCCTTGGGCACTGTAAACAGATAGctcgag
ScoC3-like-2a  271   TCCAGGGAAAGTCCTTGAACAGTGTTAATAGATAGctcgag
ScoC3-like-3a  271   CAGAGTTGGAAGGAAAGTCACTGAATCGGAGAAATCGCTAGctcgag

                                                Stop codon
```

Supplementary Figure 6- Nucleotide sequence of three ScoC3-like-a protein subunits for expression as recombinant proteins. A multiple sequence alignment of the nucleotide sequences of the three protein subunits is represented. The three ScoC3-like-a protein subunits were synthesized commercially and designed to be N-terminal His-tagged when expressed as proteins and the gene sequence was cloned into an NcoI and XhoI digested pET28a vector.

Supplementary figure 7

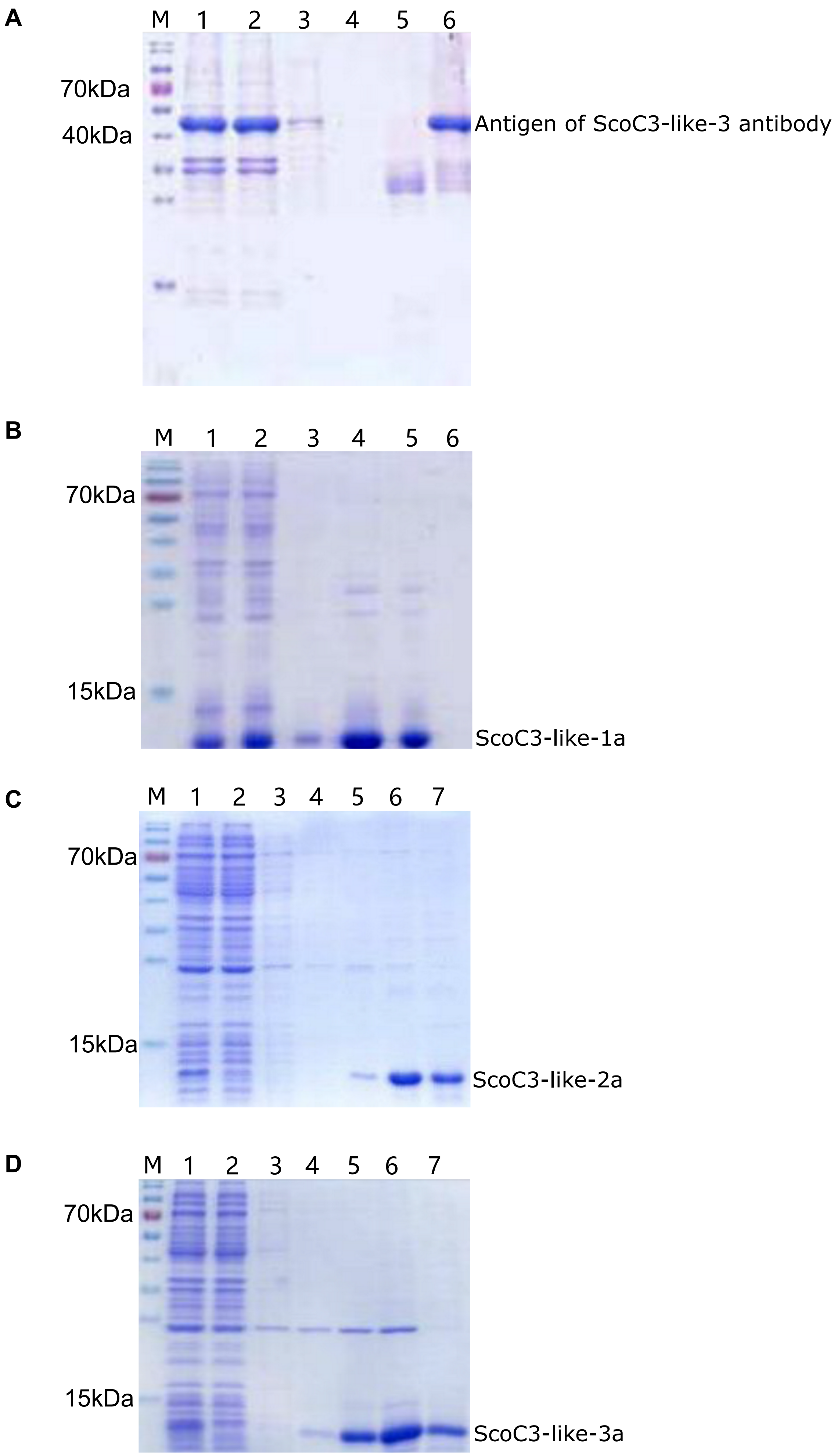

Supplementary Figure 7- SDS-PAGE gel images of the protein profile of the purified recombinant C3-like-3-b-subunit (A) and the C3-like a-subunits (B, C, D). A) Purification of the antigen amino acid sequence fragment to generate the anti-ScoC3-like-3 polyclonal sera. Antigen peptide was purified by affinity chromatography using a nitriloacetic acid (NTA) column (GE Healthcare, USA) and sample was eluted with NTAU-X (50mM PBS, pH 7.4, 0.5M NaCl, 8M Urea and X mM Imidazole, where X represents different concentration tested from 0 to 200mM). In the gels: M is the protein molecular weight marker and 1-6 corresponds to the sample fractions eluted with the grading concentration of Imidazole: NTAU-0, NTAU-10, NTAU-20, NTAU-50 and NTAU-200, respectively. In B, C and D represent the SDS-PAGE gels of eluted samples of the ScoC3-like-a recombinant protein subunits, ScoC3-like-1a (B), ScoC3-like-2a (C) and ScoC3-like-3a (D). The a-protein subunits were purified as described above for A) using NTAU-X as the eluent (where X represent Imidazole concentrations from 0 to 500 mM). In the gel: M is the protein molecular weight ladder and 1-7 correspond to the eluted samples with the different elution buffers NTAU-0, NTAU-10, NTAU-20, NTAU-50, NTAU-200 and NTAU-500, respectively
